# Supplementary material for: Modulation of Cellular Senescence in HEK293 and HepG2 Cells by Ultrafiltrates UPla and ULu Is Partly Mediated by Modulation of Mitochondrial Homeostasis under Oxidative Stress
Source: Int J Mol Sci. 2023 Apr 4;24(7):6748. doi: 10.3390/ijms24076748 (PMC10095350; doi:10.3390/ijms24076748)
Supplement: Supplementary file 1 [file ijms-24-06748-s001.zip › Tables S1 and S2.pdf]

Table S1. Proteins identified by LC-MS/MS with Mascot for UPla.

Family	Member	database	Accession	Score	emPAI	Mass	Sequence coverage	Num. of significant matches	Num. of significant unique sequences	description
3	1	Custom	sp|P02057|HBB_RABIT	25201	42.73	16179	0.86	634	7	Hemoglobin subunit beta-1/2 OS=Oryctolagus cuniculus OX=9986 GN=HBB1 PE=1 SV=2
3	2	Custom	tr|A0A5F9D756|A0A5F9D756_RABIT	22568	39.66	16547	0.88	676	7	Hemoglobin subunit gamma OS=Oryctolagus cuniculus OX=9986 PE=3 SV=1
11	1	Custom	sp|P01948|HBA_RABIT	6017	15.28	15636	0.89	232	11	Hemoglobin subunit alpha-1/2 OS=Oryctolagus cuniculus OX=9986 PE=1 SV=2
13	1	Custom	sp|Q9TTC6|PPIA_RABIT	5685	10.34	18054	0.75	155	11	Peptidyl-prolyl cis-trans isomerase A OS=Oryctolagus cuniculus OX=9986 GN=PPIA PE=2 SV=3
1	1	Custom	tr|G1U9S2|G1U9S2_RABIT	69369	10.29	70916	0.81	1894	44	Albumin OS=Oryctolagus cuniculus OX=9986 GN=ALB PE=1 SV=1
40	1	Custom	tr|G1SER8|G1SER8_RABIT	1660	6.42	11404	0.69	40	2	Profilin OS=Oryctolagus cuniculus OX=9986 GN=PFN1 PE=3 SV=2
33	1	Custom	tr|G1SZ91|G1SZ91_RABIT	2175	6.16	11636	0.76	88	5	Fatty acid binding protein 5 OS=Oryctolagus cuniculus OX=9986 GN=FABP5 PE=3 SV=3
46	1	Custom	tr|A0A5F9CP41|A0A5F9CP41_RABIT	1324	6.01	8689	0.69	53	5	Ubiquitin-like domain-containing protein OS=Oryctolagus cuniculus OX=9986 PE=4 SV=1
4	1	Custom	tr|G1STF7|G1STF7_RABIT	23012	4.81	78916	0.59	676	42	Beta-1 metal-binding globulin OS=Oryctolagus cuniculus OX=9986 PE=3 SV=1
2	1	Custom	tr|A0A5F9D486|A0A5F9D486_RABIT	26130	4.48	70926	0.76	720	35	Alpha fetoprotein OS=Oryctolagus cuniculus OX=9986 GN=AFP PE=4 SV=1
6	1	Custom	tr|A0A5F9D287|A0A5F9D287_RABIT	11441	3.94	48814	0.66	273	18	2-phospho-D-glycerate hydro-lyase OS=Oryctolagus cuniculus OX=9986 PE=3 SV=1
7	1	Custom	tr|G1THZ6|G1THZ6_RABIT	10316	3.94	37369	0.68	349	2	Immunoglobulin heavy constant mu OS=Oryctolagus cuniculus OX=9986 GN=IGHM PE=4 SV=3
7	2	Custom	sp|P01870|IGHG_RABIT	8346	3.84	35952	0.71	307	1	Ig gamma chain C region OS=Oryctolagus cuniculus OX=9986 PE=1 SV=1
10	1	Custom	sp|P00939|TPIS_RABIT	6139	3.18	27025	0.69	137	12	Triosephosphate isomerase OS=Oryctolagus cuniculus OX=9986 GN=TPI1 PE=1 SV=3
8	1	Custom	sp|P21195|PDIA1_RABIT	9937	3.14	57172	0.58	328	24	Protein disulfide-isomerase OS=Oryctolagus cuniculus OX=9986 GN=P4HB PE=2 SV=1

32	1	Custom	tr|A0A5F9C6Q3|A0A5F9C6Q3_RABIT	2271	2.56	13607	0.73	63	6	Transthyretin OS=Oryctolagus cuniculus OX=9986 GN=TTR PE=3 SV=1
19	1	Custom	sp|P00919|CAH2_RABIT	3803	2.33	29596	0.57	113	12	Carbonic anhydrase 2 OS=Oryctolagus cuniculus OX=9986 GN=CA2 PE=1 SV=3
51	1	Custom	tr|G1SPF5|G1SPF5_RABIT	1194	2.31	14574	0.71	48	4	Phosphoglycerate mutase 1 OS=Oryctolagus cuniculus OX=9986 GN=PGAM1 PE=4 SV=2
40	2	contaminants	P02584	1109	2.15	15219	0.41	32	1	SWISS-PROT:P02584 (Bos taurus) Profilin-1
5	1	Custom	tr|G1T9M9|G1T9M9_RABIT	11875	2.15	71082	0.5	293	23	Heat shock protein family A (Hsp70) member 8 OS=Oryctolagus cuniculus OX=9986 PE=3 SV=2
14	1	Custom	tr|A0A5F9C4B7|A0A5F9C4B7_RABIT	5340	2.14	28572	0.45	124	8	Tyrosine 3-monooxygenase/tryptophan 5-monooxygenase activation protein zeta OS=Oryctolagus cuniculus OX=9986 GN=YWHAZ PE=3 SV=1
12	1	Custom	tr|U3KMR2|U3KMR2_RABIT	5858	2.12	36689	0.53	149	12	Alpha-2-HS-glycoprotein OS=Oryctolagus cuniculus OX=9986 GN=AHSG PE=4 SV=1
9	3	Custom	sp|P23035|A1AF_RABIT	6786	2.02	46010	0.48	174	1	Alpha-1-antiproteinase F OS=Oryctolagus cuniculus OX=9986 PE=1 SV=1
17	1	Custom	tr|G1TV43|G1TV43_RABIT	4887	1.93	41753	0.58	156	15	Aldo-keto reductase family 1 member B10 OS=Oryctolagus cuniculus OX=9986 GN=AKR1B10 PE=3 SV=2
9	2	Custom	tr|G1TFV7|G1TFV7_RABIT	8784	1.89	50899	0.47	215	0	SERPIN domain-containing protein OS=Oryctolagus cuniculus OX=9986 GN=SERPINA1 PE=3 SV=2
16	1	Custom	sp|P15253|CALR_RABIT	4896	1.87	48416	0.58	198	17	Calreticulin OS=Oryctolagus cuniculus OX=9986 GN=CALR PE=1 SV=1
23	1	Custom	sp|P01840|KAC4_RABIT	3308	1.78	11264	0.63	95	3	Ig kappa-b4 chain C region OS=Oryctolagus cuniculus OX=9986 PE=1 SV=1
44	1	Custom	tr|G1U7U3|G1U7U3_RABIT	1337	1.75	17315	0.41	62	0	Nucleoside diphosphate kinase OS=Oryctolagus cuniculus OX=9986 GN=NME1 PE=3 SV=2
5	5	Custom	tr|A0A5F9C804|A0A5F9C804_RABIT	9294	1.75	71475	0.47	229	22	78 kDa glucose-regulated protein OS=Oryctolagus cuniculus OX=9986 GN=HSPA5 PE=3 SV=1
9	1	Custom	tr|G1TFX2|G1TFX2_RABIT	8858	1.73	50843	0.44	209	0	SERPIN domain-containing protein OS=Oryctolagus cuniculus OX=9986 GN=SERPINA1 PE=3 SV=2
21	1	Custom	tr|A0A5S8H3Z9|A0A5S8H3Z9_RABIT	3343	1.68	36269	0.34	90	10	Alpha-1-acid glycoprotein OS=Oryctolagus cuniculus OX=9986 PE=3 SV=1
49	1	Custom	tr|A0A5F9CQ70|A0A5F9CQ70_RABIT	1254	1.65	24329	0.44	50	8	Thioredoxin domain-containing protein OS=Oryctolagus cuniculus OX=9986 PE=3 SV=1
35	1	contaminants	P00761	2086	1.57	25078	0.25	94	4	SWISS-PROT:P00761|TRYP_PIG Trypsin - Sus scrofa (Pig).
14	3	Custom	tr|G1SZD6|G1SZD6_RABIT	1952	1.33	28044	0.26	55	4	14-3-3 protein theta OS=Oryctolagus cuniculus OX=9986 GN=YWHAQ PE=3 SV=1

44	2	Custom	tr|U3KNZ4|U3KNZ4_RABIT	1300	1.31	24747	0.35	64	2	Nucleoside diphosphate kinase OS=Oryctolagus cuniculus OX=9986 PE=3 SV=1
14	2	Custom	tr|A0A5F9CVU0|A0A5F9CVU0_RABIT	3211	1.26	47748	0.27	107	10	Tyrosine 3-monooxygenase/tryptophan 5-monooxygenase activation protein epsilon OS=Oryctolagus cuniculus OX=9986 GN=YWHAE PE=3 SV=1
34	3	Custom	tr|G1SXP3|G1SXP3_RABIT	1106	1.25	10559	0.38	31	1	GST C-terminal domain-containing protein OS=Oryctolagus cuniculus OX=9986 PE=4 SV=2
34	2	Custom	tr|A0A5F9CJ08|A0A5F9CJ08_RABIT	2022	1.25	25643	0.41	59	1	Glutathione S-transferase OS=Oryctolagus cuniculus OX=9986 GN=GSTM2 PE=3 SV=1
50	1	Custom	tr|G1SQ02|G1SQ02_RABIT	1232	1.21	22366	0.45	37	6	Peroxiredoxin-1 OS=Oryctolagus cuniculus OX=9986 PE=3 SV=1
9	4	Custom	tr|G1T809|G1T809_RABIT	3695	1.14	51248	0.32	93	3	Serpin family A member 9 OS=Oryctolagus cuniculus OX=9986 GN=SERPINA9 PE=3 SV=1
63	1	Custom	sp|P03984|KAC6_RABIT	650	1.13	11300	0.68	16	3	Ig kappa chain b5 variant C region OS=Oryctolagus cuniculus OX=9986 PE=2 SV=1
48	1	Custom	sp|P24480|S10AB_RABIT	1298	1.11	11536	0.33	28	3	Protein S100-A11 OS=Oryctolagus cuniculus OX=9986 GN=S100A11 PE=1 SV=2
14	4	Custom	tr|G1TZP0|G1TZP0_RABIT	1815	1.08	28412	0.28	54	2	Tyrosine 3-monooxygenase/tryptophan 5-monooxygenase activation protein gamma OS=Oryctolagus cuniculus OX=9986 GN=YWHAG PE=3 SV=1
15	1	Custom	tr|G1ST29|G1ST29_RABIT	4949	1.06	28895	0.27	95	7	Myelin P2 protein OS=Oryctolagus cuniculus OX=9986 PE=3 SV=2
55	1	Custom	tr|A0A5F9CW87|A0A5F9CW87_RABIT	957	1.05	12038	0.32	21	3	Thioredoxin OS=Oryctolagus cuniculus OX=9986 PE=3 SV=1
43	2	Custom	tr|G1TMP1|G1TMP1_RABIT	1339	1.02	46988	0.31	48	8	IF rod domain-containing protein OS=Oryctolagus cuniculus OX=9986 PE=4 SV=2
77	1	Custom	sp|P01697|KV16_RABIT	471	1.01	12276	0.3	13	1	Ig kappa chain V region AH80-5 OS=Oryctolagus cuniculus OX=9986 PE=1 SV=1
25	1	Custom	tr|G1TVS4|G1TVS4_RABIT	3231	0.99	52317	0.33	122	12	Hemopexin OS=Oryctolagus cuniculus OX=9986 GN=TRIM3 PE=3 SV=2
34	1	Custom	tr|A0A5F9D4U8|A0A5F9D4U8_RABIT	2126	0.98	30450	0.34	62	1	Glutathione S-transferase OS=Oryctolagus cuniculus OX=9986 GN=GSTM3 PE=3 SV=1
28	1	Custom	tr|A0A5F9CNW6|A0A5F9CNW6_RABIT	2827	0.94	59107	0.33	71	12	Glucose-6-phosphate isomerase OS=Oryctolagus cuniculus OX=9986 GN=GPI PE=3 SV=1
36	1	Custom	tr|G1SQG5|G1SQG5_RABIT	2024	0.92	36643	0.39	67	8	Malate dehydrogenase OS=Oryctolagus cuniculus OX=9986 GN=MDH1 PE=3 SV=1
29	1	contaminants	P60712	2602	0.9	42052	0.42	74	7	SWISS-PROT:P60712 (Bos taurus) Actin, cytoplasmic 1

96	1	Custom	tr|G1TME7|G1TME7_RABIT	255	0.87	9026	0.35	12	2	Cystatin B OS=Oryctolagus cuniculus OX=9986 GN=CSTB PE=3 SV=1
20	1	Custom	tr|A0A5F9CCJ1|A0A5F9CCJ1_RABIT	3553	0.85	63725	0.33	117	13	Gc-globulin OS=Oryctolagus cuniculus OX=9986 GN=GC PE=4 SV=1
30	2	Custom	tr|G1SS18|G1SS18_RABIT	2288	0.84	54172	0.23	64	10	IF rod domain-containing protein OS=Oryctolagus cuniculus OX=9986 PE=3 SV=2
44	3	Custom	tr|A0A5F9DFZ0|A0A5F9DFZ0_RABIT	557	0.81	9535	0.22	13	1	NDK domain-containing protein OS=Oryctolagus cuniculus OX=9986 PE=3 SV=1
41	1	Custom	tr|A0A5F9D5U7|A0A5F9D5U7_RABIT	1604	0.81	45609	0.29	61	7	Fructose-bisphosphate aldolase OS=Oryctolagus cuniculus OX=9986 PE=3 SV=1
73	1	Custom	tr|G1T7R1|G1T7R1_RABIT	529	0.8	14806	0.25	20	3	Fatty acid binding protein 3 OS=Oryctolagus cuniculus OX=9986 GN=FABP3 PE=3 SV=1
53	1	Custom	tr|G1SZV5|G1SZV5_RABIT	1070	0.79	30615	0.29	31	6	Apolipoprotein A-I OS=Oryctolagus cuniculus OX=9986 GN=APOA1 PE=3 SV=1
38	1	Custom	sp|P07452|CAH1_RABIT	1832	0.78	25737	0.31	37	5	Carbonic anhydrase 1 (Fragment) OS=Oryctolagus cuniculus OX=9986 GN=CA1 PE=2 SV=1
95	1	Custom	tr|G1TKH3|G1TKH3_RABIT	267	0.74	15773	0.55	15	3	Superoxide dismutase [Cu-Zn] OS=Oryctolagus cuniculus OX=9986 GN=SOD1 PE=3 SV=1
18	2	Custom	tr|A0A5F9D4S1|A0A5F9D4S1_RABIT	3726	0.71	73305	0.3	88	7	Actinin alpha 4 OS=Oryctolagus cuniculus OX=9986 GN=ACTN4 PE=4 SV=1
78	1	Custom	tr|G1T2C4|G1T2C4_RABIT	459	0.68	22685	0.23	11	4	Transgelin OS=Oryctolagus cuniculus OX=9986 PE=3 SV=2
42	1	Custom	tr|G1T804|G1T804_RABIT	1535	0.68	34578	0.31	29	6	Uncharacterized protein OS=Oryctolagus cuniculus OX=9986 PE=4 SV=2
70	1	Custom	tr|A0A5F9CII3|A0A5F9CII3_RABIT	553	0.67	28849	0.17	17	1	Chromosome 1 open reading frame 43 OS=Oryctolagus cuniculus OX=9986 GN=C1orf43 PE=3 SV=1
14	5	Custom	tr|A0A5F9CN85|A0A5F9CN85_RABIT	1472	0.67	41097	0.16	53	2	Tyrosine 3-monooxygenase/tryptophan 5-monooxygenase activation protein beta OS=Oryctolagus cuniculus OX=9986 GN=YWHAB PE=3 SV=1
58	1	Custom	tr|G1SIK0|G1SIK0_RABIT	842	0.66	53059	0.28	26	9	Antithrombin-III OS=Oryctolagus cuniculus OX=9986 GN=SERPINC1 PE=3 SV=1
6	2	Custom	sp|P25704|ENOB_RABIT	4377	0.66	47381	0.29	86	3	Beta-enolase OS=Oryctolagus cuniculus OX=9986 GN=ENO3 PE=1 SV=4
37	1	Custom	tr|A0A5F9CFP1|A0A5F9CFP1_RABIT	1889	0.65	53675	0.33	61	9	Alpha-1B-glycoprotein OS=Oryctolagus cuniculus OX=9986 GN=A1BG PE=4 SV=1
66	1	Custom	tr|G1TKA1|G1TKA1_RABIT	400	0.64	11558	0.14	18	1	Ig-like domain-containing protein OS=Oryctolagus cuniculus OX=9986 PE=4 SV=2

77	2	Custom	sp|P01696|KV15_RABIT	241	0.63	11760	0.19	6	1	Ig kappa chain V region K29-213 OS=Oryctolagus cuniculus OX=9986 PE=1 SV=1
77	3	Custom	sp|P01683|KV02_RABIT	235	0.6	12233	0.21	6	1	Ig kappa chain V region 3315 OS=Oryctolagus cuniculus OX=9986 PE=1 SV=1
39	1	Custom	tr|A0A5F9CD26|A0A5F9CD26_RABIT	1751	0.58	39212	0.22	57	6	Haptoglobin OS=Oryctolagus cuniculus OX=9986 PE=3 SV=1
24	1	Custom	tr|G1TBC1|G1TBC1_RABIT	3256	0.58	92618	0.23	85	14	Endoplasmin OS=Oryctolagus cuniculus OX=9986 GN=HSP90B1 PE=3 SV=2
43	3	contaminants	P13645	1309	0.57	59703	0.18	37	6	SWISS-PROT:P13645 Tax_Id=9606 Gene_Symbol=KRT10 Keratin, type I cytoskeletal 10
52	3	Custom	tr|G1SQE0|G1SQE0_RABIT	506	0.57	12772	0.22	11	1	Ig-like domain-containing protein OS=Oryctolagus cuniculus OX=9986 PE=4 SV=3
70	3	Custom	tr|A0A5F9DP16|A0A5F9DP16_RABIT	397	0.57	33129	0.13	14	1	Chromosome 1 open reading frame 43 OS=Oryctolagus cuniculus OX=9986 GN=C1orf43 PE=3 SV=1
27	1	Custom	tr|G1SWS9|G1SWS9_RABIT	3122	0.56	53679	0.18	69	8	Vimentin OS=Oryctolagus cuniculus OX=9986 GN=VIM PE=3 SV=1
64	1	Custom	tr|G1TBS1|G1TBS1_RABIT	637	0.55	20098	0.17	16	3	Maillard deglycase OS=Oryctolagus cuniculus OX=9986 GN=PARK7 PE=3 SV=1
68	1	Custom	tr|A0A5F9CKK9|A0A5F9CKK9_RABIT	561	0.55	34235	0.36	16	5	Biliverdin reductase B OS=Oryctolagus cuniculus OX=9986 GN=BLVRB PE=4 SV=1
93	1	Custom	tr|A0A5F9CDD8|A0A5F9CDD8_RABIT	284	0.53	28123	0.29	10	4	Peroxiredoxin 4 OS=Oryctolagus cuniculus OX=9986 GN=PRDX4 PE=4 SV=1
18	1	Custom	tr|A0A5F9C1L6|A0A5F9C1L6_RABIT	4352	0.5	97463	0.19	94	7	Actinin alpha 1 OS=Oryctolagus cuniculus OX=9986 GN=ACTN1 PE=4 SV=1
70	2	Custom	tr|A0A5F9DVE1|A0A5F9DVE1_RABIT	479	0.49	37548	0.13	18	3	Tropomyosin 4 OS=Oryctolagus cuniculus OX=9986 GN=TPM4 PE=3 SV=1
1	3	contaminants	P02769	2401	0.46	71244	0.12	107	2	SWISS-PROT:P02769 (Bos taurus) Bovine serum albumin precursor
45	1	Custom	tr|A0A5F9CY41|A0A5F9CY41_RABIT	1329	0.46	55978	0.21	38	7	Uncharacterized protein OS=Oryctolagus cuniculus OX=9986 PE=3 SV=1
61	1	Custom	tr|G1SFV1|G1SFV1_RABIT	757	0.45	73058	0.15	33	9	Protein disulfide-isomerase OS=Oryctolagus cuniculus OX=9986 GN=PDIA4 PE=3 SV=2
30	1	contaminants	P04264	2571	0.44	66149	0.15	53	7	SWISS-PROT:P04264 Tax_Id=9606 Gene_Symbol=KRT1 Keratin, type II cytoskeletal 1

43	4	Custom	tr|A0A5F9CRZ7|A0A5F9CRZ7_RABIT	874	0.44	49870	0.15	31	3	Keratin 15 OS=Oryctolagus cuniculus OX=9986 GN=KRT15 PE=3 SV=1
52	1	Custom	tr|G1TK85|G1TK85_RABIT	1109	0.43	41559	0.2	30	1	Immunoglobulin heavy constant mu OS=Oryctolagus cuniculus OX=9986 GN=IGHM PE=4 SV=2
22	1	Custom	tr|A0A5F9C343|A0A5F9C343_RABIT	3337	0.43	92846	0.2	80	10	Periostin OS=Oryctolagus cuniculus OX=9986 GN=POSTN PE=4 SV=1
173	1	Custom	tr|G1T3Q4|G1T3Q4_RABIT	32	0.42	16741	0.26	2	2	Calmodulin like 5 OS=Oryctolagus cuniculus OX=9986 GN=CALML5 PE=4 SV=1
65	1	Custom	tr|A0A5F9CTP7|A0A5F9CTP7_RABIT	609	0.41	43206	0.12	24	5	Fetuin B OS=Oryctolagus cuniculus OX=9986 GN=FETUB PE=4 SV=1
86	1	Custom	tr|A0A5F9C975|A0A5F9C975_RABIT	391	0.41	16837	0.1	15	1	Ig-like domain-containing protein OS=Oryctolagus cuniculus OX=9986 PE=4 SV=1
60	1	Custom	sp|P62160|CALM_RABIT	774	0.41	16827	0.22	21	2	Calmodulin OS=Oryctolagus cuniculus OX=9986 GN=CALM PE=1 SV=2
146	1	Custom	tr|A0A5F9CXV9|A0A5F9CXV9_RABIT	54	0.41	8163	0.17	1	1	Rad60-SLD domain-containing protein OS=Oryctolagus cuniculus OX=9986 PE=4 SV=1
26	1	Custom	tr|A0A5F9CAH6|A0A5F9CAH6_RABIT	3163	0.4	162896	0.21	104	17	Alpha-2-macroglobulin OS=Oryctolagus cuniculus OX=9986 GN=A2M PE=3 SV=1
75	1	Custom	tr|B7NZF1|B7NZF1_RABIT	483	0.37	56671	0.17	19	6	Protein disulfide-isomerase OS=Oryctolagus cuniculus OX=9986 GN=PDIA3 PE=3 SV=1
51	2	Custom	tr|G1U7S4|G1U7S4_RABIT	262	0.36	28820	0.21	15	2	Phosphoglycerate mutase OS=Oryctolagus cuniculus OX=9986 GN=PGAM2 PE=1 SV=1
52	2	Custom	sp|P03988|IGHM_RABIT	662	0.35	50550	0.16	21	2	Ig mu chain C region secreted form OS=Oryctolagus cuniculus OX=9986 PE=2 SV=1
1	2	contaminants	P02768-1	5970	0.34	71317	0.08	264	1	SWISS-PROT:P02768-1 Tax_Id=9606 Gene_Symbol=ALB Isoform 1 of Serum albumin precursor
24	2	Custom	tr|A0A0G2JH20|A0A0G2JH20_RABIT	1458	0.34	81713	0.13	43	8	Heat shock protein 90 alpha family class A member 1 OS=Oryctolagus cuniculus OX=9986 PE=3 SV=2
43	1	contaminants	P35527	1451	0.34	62320	0.17	48	5	SWISS-PROT:P35527 Tax_Id=9606 Gene_Symbol=KRT9 Keratin, type I cytoskeletal 9
172	1	Custom	tr|A0A5F9D1U9|A0A5F9D1U9_RABIT	32	0.33	20403	0.3	2	2	TCTP domain-containing protein OS=Oryctolagus cuniculus OX=9986 PE=3 SV=1
84	1	Custom	sp|Q8WN94|ACBP_RABIT	402	0.33	9909	0.11	16	1	Acyl-CoA-binding protein OS=Oryctolagus cuniculus OX=9986 GN=DBI PE=3 SV=3

74	1	Custom	tr|A0A5F9C8E0|A0A5F9C8E0_RABIT	504	0.33	52778	0.14	13	5	Aldo-keto reductase family 1 member A1 OS=Oryctolagus cuniculus OX=9986 GN=AKR1A1 PE=3 SV=1
34	4	Custom	tr|G1SXQ0|G1SXQ0_RABIT	468	0.31	33150	0.12	12	3	Glutathione S-transferase OS=Oryctolagus cuniculus OX=9986 GN=GSTM3 PE=3 SV=2
66	2	Custom	tr|G1TSJ0|G1TSJ0_RABIT	56	0.31	22138	0.08	4	1	Ig-like domain-containing protein OS=Oryctolagus cuniculus OX=9986 PE=4 SV=2
52	4	Custom	tr|G1TGX7|G1TGX7_RABIT	505	0.3	22314	0.12	13	1	Ig-like domain-containing protein OS=Oryctolagus cuniculus OX=9986 PE=4 SV=3
82	2	Custom	tr|A0A5F9DK78|A0A5F9DK78_RABIT	357	0.3	45875	0.1	16	1	Rab GDP dissociation inhibitor OS=Oryctolagus cuniculus OX=9986 PE=3 SV=1
74	2	Custom	sp|P15122|ALDR_RABIT	111	0.28	36140	0.08	8	3	Aldo-keto reductase family 1 member B1 OS=Oryctolagus cuniculus OX=9986 GN=AKR1B1 PE=2 SV=3
82	1	Custom	tr|G1TE41|G1TE41_RABIT	429	0.28	48611	0.14	18	1	Rab GDP dissociation inhibitor OS=Oryctolagus cuniculus OX=9986 GN=GDI1 PE=3 SV=1
62	1	Custom	tr|A0A5F9DVP8|A0A5F9DVP8_RABIT	723	0.28	24328	0.13	14	2	Retinol-binding protein OS=Oryctolagus cuniculus OX=9986 PE=3 SV=1
115	1	Custom	sp|P62943|FKB1A_RABIT	137	0.27	12000	0.12	5	1	Peptidyl-prolyl cis-trans isomerase FKBP1A OS=Oryctolagus cuniculus OX=9986 GN=FKBP1A PE=1 SV=2
105	1	Custom	tr|A0A5F9D447|A0A5F9D447_RABIT	171	0.27	12048	0.09	4	1	Beta-2-microglobulin OS=Oryctolagus cuniculus OX=9986 GN=B2M PE=4 SV=1
31	1	Custom	tr|G1SP97|G1SP97_RABIT	2330	0.26	38736	0.12	54	3	Lumican OS=Oryctolagus cuniculus OX=9986 GN=LUM PE=3 SV=1
215	1	Custom	tr|G1T6M1|G1T6M1_RABIT	21	0.26	12491	0.07	1	1	DnaJ heat shock protein family (Hsp40) member C19 OS=Oryctolagus cuniculus OX=9986 GN=DNAJC19 PE=4 SV=1
41	2	Custom	tr|G1T652|G1T652_RABIT	329	0.25	39735	0.14	12	1	Fructose-bisphosphate aldolase OS=Oryctolagus cuniculus OX=9986 GN=ALDOC PE=3 SV=1
118	1	Custom	tr|A0A5F9C6K5|A0A5F9C6K5_RABIT	118	0.25	40111	0.18	3	3	Hydroxyacyl-CoA dehydrogenase OS=Oryctolagus cuniculus OX=9986 GN=HADH PE=3 SV=1
56	1	Custom	tr|A0A5F9D4A7|A0A5F9D4A7_RABIT	943	0.25	53676	0.15	21	4	SERPIN domain-containing protein OS=Oryctolagus cuniculus OX=9986 GN=SERPINF2 PE=3 SV=1
136	1	Custom	tr|G1T670|G1T670_RABIT	74	0.25	26549	0.12	3	2	Proteasome subunit alpha type OS=Oryctolagus cuniculus OX=9986 GN=PSMA5 PE=3 SV=2
29	2	Custom	tr|G1SDT0|G1SDT0_RABIT	915	0.24	41913	0.17	23	1	Actin beta like 2 OS=Oryctolagus cuniculus OX=9986 GN=ACTBL2 PE=3 SV=2
176	1	Custom	tr|A0A5F9CQP0|A0A5F9CQP0_RABIT	30	0.23	13958	0.06	3	1	GLOBIN domain-containing protein OS=Oryctolagus cuniculus OX=9986 PE=3 SV=1

154	1	Custom	tr|A0A5F9DJ89|A0A5F9DJ89_RABIT	46	0.22	14498	0.12	2	1	2-iminobutanoate/2-iminopropanoate deaminase OS=Oryctolagus cuniculus OX=9986 PE=3 SV=1
79	1	Custom	tr|G1TM88|G1TM88_RABIT	459	0.22	46013	0.1	16	3	Serpin family A member 3 OS=Oryctolagus cuniculus OX=9986 GN=SERPINA3 PE=3 SV=2
207	1	Custom	tr|G1TPZ1|G1TPZ1_RABIT	23	0.22	15170	0.14	1	1	Galectin OS=Oryctolagus cuniculus OX=9986 GN=LGALS1 PE=4 SV=1
90	1	Custom	tr|G1TIY2|G1TIY2_RABIT	372	0.19	16645	0.07	10	1	Ig-like domain-containing protein OS=Oryctolagus cuniculus OX=9986 PE=4 SV=3
121	1	Custom	tr|G1TJR8|G1TJR8_RABIT	111	0.19	16649	0.08	12	1	Ig-like domain-containing protein OS=Oryctolagus cuniculus OX=9986 PE=4 SV=2
185	1	Custom	tr|A0A5F9D468|A0A5F9D468_RABIT	28	0.18	17263	0.04	2	1	Ring finger protein 185 OS=Oryctolagus cuniculus OX=9986 GN=RNF185 PE=4 SV=1
133	1	Custom	tr|A0A5F9CC29|A0A5F9CC29_RABIT	81	0.18	17270	0.1	5	1	Ig-like domain-containing protein OS=Oryctolagus cuniculus OX=9986 PE=4 SV=1
91	1	Custom	tr|A0A5F9CBR3|A0A5F9CBR3_RABIT	314	0.18	35454	0.14	7	2	Aldo_ket_red domain-containing protein OS=Oryctolagus cuniculus OX=9986 PE=3 SV=1
186	1	Custom	tr|A0A5F9C2D7|A0A5F9C2D7_RABIT	28	0.17	18495	0.05	2	1	Uncharacterized protein OS=Oryctolagus cuniculus OX=9986 PE=4 SV=1
129	1	Custom	tr|A0A5F9CRP9|A0A5F9CRP9_RABIT	92	0.17	18709	0.12	3	1	Uncharacterized protein OS=Oryctolagus cuniculus OX=9986 PE=3 SV=1
166	1	Custom	tr|A0A5F9CB49|A0A5F9CB49_RABIT	38	0.17	18898	0.27	1	1	TCTP domain-containing protein OS=Oryctolagus cuniculus OX=9986 PE=3 SV=1
107	1	Custom	tr|A0A5F9D5A4|A0A5F9D5A4_RABIT	165	0.17	19235	0.09	3	1	RAB1A, member RAS oncogene family OS=Oryctolagus cuniculus OX=9986 GN=RAB1A PE=4 SV=1
87	1	Custom	tr|A0A5F9CPB1|A0A5F9CPB1_RABIT	384	0.16	19746	0.09	5	1	GST class-pi OS=Oryctolagus cuniculus OX=9986 PE=3 SV=1
116	1	Custom	tr|A0A5F9CQR3|A0A5F9CQR3_RABIT	127	0.16	19766	0.16	3	1	Uncharacterized protein OS=Oryctolagus cuniculus OX=9986 PE=3 SV=1
57	1	Custom	tr|G1STJ4|G1STJ4_RABIT	905	0.16	82945	0.08	18	4	Complement C3 alpha chain OS=Oryctolagus cuniculus OX=9986 PE=4 SV=3
59	1	Custom	tr|G1TKE3|G1TKE3_RABIT	836	0.15	20402	0.1	10	1	C1q domain-containing protein OS=Oryctolagus cuniculus OX=9986 PE=4 SV=2

72	1	Custom	tr|G1TZA1|G1TZA1_RABIT	533	0.15	20724	0.08	11	1	Mediator of RNA polymerase II transcription subunit 6 OS=Oryctolagus cuniculus OX=9986 GN=MED6 PE=3 SV=1
122	2	Custom	tr|A0A5F9DAJ6|A0A5F9DAJ6_RABIT	95	0.15	21122	0.04	10	1	Phosphatidylethanolamine-binding protein 1 OS=Oryctolagus cuniculus OX=9986 GN=PEBP1 PE=3 SV=2
47	1	Custom	tr|G1SV22|G1SV22_RABIT	1300	0.15	21110	0.11	15	1	Transgelin OS=Oryctolagus cuniculus OX=9986 GN=TAGLN2 PE=3 SV=2
141	1	Custom	tr|G1TAB2|G1TAB2_RABIT	62	0.15	21252	0.1	1	1	ML domain-containing protein OS=Oryctolagus cuniculus OX=9986 PE=4 SV=1
30	3	contaminants	P35908	894	0.15	66110	0.1	25	1	SWISS-PROT:P35908 Tax_Id=9606 Gene_Symbol=KRT2 Keratin, type II cytoskeletal 2 epidermal
113	1	Custom	tr|A0A5F9C1I9|A0A5F9C1I9_RABIT	138	0.15	44156	0.05	18	2	Actin-like protein 3 OS=Oryctolagus cuniculus OX=9986 PE=3 SV=1
92	1	Custom	tr|G1T7Z6|G1T7Z6_RABIT	295	0.14	45003	0.08	8	2	Phosphoglycerate kinase OS=Oryctolagus cuniculus OX=9986 PE=3 SV=2
101	1	Custom	tr|A0A5F9DEW0|A0A5F9DEW0_RABIT	185	0.14	22419	0.06	2	1	Heme binding protein 2 OS=Oryctolagus cuniculus OX=9986 GN=HEBP2 PE=3 SV=1
138	1	Custom	tr|G1T4X8|G1T4X8_RABIT	70	0.14	22980	0.09	4	1	Proteasome subunit beta OS=Oryctolagus cuniculus OX=9986 GN=PSMB2 PE=3 SV=2
112	1	Custom	tr|G1T332|G1T332_RABIT	141	0.14	46715	0.09	3	2	Aspartate aminotransferase OS=Oryctolagus cuniculus OX=9986 GN=GOT1 PE=3 SV=1
128	1	Custom	tr|A0A5F9C295|A0A5F9C295_RABIT	97	0.14	23078	0.06	1	1	Proteasome subunit alpha type OS=Oryctolagus cuniculus OX=9986 GN=PSMA6 PE=3 SV=1
69	1	Custom	tr|G1TRY5|G1TRY5_RABIT	560	0.14	71307	0.09	9	3	Plastin 3 OS=Oryctolagus cuniculus OX=9986 GN=PLS3 PE=4 SV=2
209	1	Custom	tr|A0A5F9CY61|A0A5F9CY61_RABIT	22	0.13	23433	0.04	1	1	Uncharacterized protein OS=Oryctolagus cuniculus OX=9986 PE=4 SV=1
132	1	Custom	tr|G1TEC1|G1TEC1_RABIT	81	0.13	23585	0.07	1	1	Ig-like domain-containing protein OS=Oryctolagus cuniculus OX=9986 PE=4 SV=2
81	1	Custom	tr|A0A5F9DR01|A0A5F9DR01_RABIT	445	0.13	48652	0.06	8	2	Aspartate aminotransferase OS=Oryctolagus cuniculus OX=9986 GN=GOT2 PE=3 SV=1
85	1	Custom	tr|G1TI72|G1TI72_RABIT	399	0.13	50108	0.02	27	1	TBC1 domain family member 10C OS=Oryctolagus cuniculus OX=9986 GN=TBC1D10C PE=4 SV=3
131	1	Custom	tr|A0A5F9CSE7|A0A5F9CSE7_RABIT	84	0.13	50211	0.06	2	2	Alpha-1-microglobulin OS=Oryctolagus cuniculus OX=9986 GN=KIF12 PE=3 SV=1
4	2	Custom	tr|G1TKE4|G1TKE4_RABIT	1019	0.12	77354	0.05	24	1	Uncharacterized protein OS=Oryctolagus cuniculus OX=9986 PE=3 SV=1

98	1	Custom	tr|G1SDD6|G1SDD6_RABIT	213	0.12	51700	0.06	3	2	Angiotensin 1-10 OS=Oryctolagus cuniculus OX=9986 GN=AGT PE=3 SV=3
80	1	Custom	tr|A0A5F9DV00|A0A5F9DV00_RABIT	454	0.12	25724	0.04	11	1	PlaK185:K198telet activating factor acetylhydrolase 1b catalytic subunit 2 OS=Oryctolagus cuniculus OX=9986 GN=PAFAH1B2 PE=4 SV=1
157	1	Custom	tr|G1TAK3|G1TAK3_RABIT	45	0.12	26275	0.06	6	1	Metalloproteinase inhibitor 4 OS=Oryctolagus cuniculus OX=9986 GN=TIMP4 PE=3 SV=1
83	1	Custom	tr|A0A5F9CDG0|A0A5F9CDG0_RABIT	415	0.12	26778	0.07	5	1	ADF-H domain-containing protein OS=Oryctolagus cuniculus OX=9986 PE=4 SV=1
139	1	Custom	tr|G1TXC0|G1TXC0_RABIT	69	0.11	27830	0.05	1	1	Caspase 14 OS=Oryctolagus cuniculus OX=9986 GN=CASP14 PE=3 SV=3
165	1	Custom	tr|G1T918|G1T918_RABIT	39	0.11	29016	0.03	1	1	Proteasome subunit beta OS=Oryctolagus cuniculus OX=9986 GN=PSMB4 PE=3 SV=1
149	1	Custom	tr|A0A5F9CHA6|A0A5F9CHA6_RABIT	51	0.11	29417	0.07	2	1	Ig-like domain-containing protein OS=Oryctolagus cuniculus OX=9986 PE=4 SV=1
216	1	Custom	tr|A0A5F9CRU2|A0A5F9CRU2_RABIT	20	0.11	29655	0.06	1	1	Short chain dehydrogenase/reductase family 16C member 5 OS=Oryctolagus cuniculus OX=9986 GN=SDR16C5 PE=4 SV=1
130	1	Custom	tr|A0A5F9C1B3|A0A5F9C1B3_RABIT	91	0.1	29865	0.04	4	1	Ig-like domain-containing protein OS=Oryctolagus cuniculus OX=9986 PE=4 SV=1
71	1	Custom	tr|G1SQG6|G1SQG6_RABIT	537	0.1	63588	0.07	10	2	SERPIN domain-containing protein OS=Oryctolagus cuniculus OX=9986 PE=3 SV=3
150	1	Custom	tr|A0A5F9DCJ0|A0A5F9DCJ0_RABIT	51	0.1	31717	0.09	2	1	Crystallin zeta OS=Oryctolagus cuniculus OX=9986 GN=CRYZ PE=3 SV=1
155	1	Custom	tr|G1SN21|G1SN21_RABIT	45	0.1	32277	0.03	1	1	Purine nucleoside phosphorylase OS=Oryctolagus cuniculus OX=9986 GN=PNP PE=3 SV=2
67	1	Custom	tr|A0A5F9CGS8|A0A5F9CGS8_RABIT	569	0.09	33284	0.04	9	1	Alpha-2-glycoprotein 1, zinc-binding OS=Oryctolagus cuniculus OX=9986 GN=AZGP1 PE=3 SV=1
135	1	Custom	tr|A0A5F9C1C3|A0A5F9C1C3_RABIT	79	0.09	33488	0.05	1	1	Thioredoxin domain-containing protein OS=Oryctolagus cuniculus OX=9986 PE=4 SV=1
89	1	Custom	tr|A0A5F9DNF9|A0A5F9DNF9_RABIT	382	0.09	102381	0.06	7	3	Aminopeptidase OS=Oryctolagus cuniculus OX=9986 GN=NPEPPS PE=3 SV=1
181	1	Custom	tr|A0A5F9C413|A0A5F9C413_RABIT	29	0.09	34916	0.03	1	1	GULP PTB domain containing engulfment adaptor 1 OS=Oryctolagus cuniculus OX=9986 GN=GULP1 PE=4 SV=1

114	1	Custom	tr|A0A5F9D3N8|A0A5F9D3N8_RABIT	138	0.09	107260	0.07	6	3	Gal_mutarotas_2 domain-containing protein OS=Oryctolagus cuniculus OX=9986 PE=3 SV=1
94	1	Custom	tr|A0A5F9CHF0|A0A5F9CHF0_RABIT	276	0.09	71209	0.04	9	2	Kininogen 1 OS=Oryctolagus cuniculus OX=9986 GN=KNG1 PE=4 SV=1
145	1	Custom	tr|G1T1V5|G1T1V5_RABIT	55	0.09	36151	0.06	3	1	Carbonic anhydrase OS=Oryctolagus cuniculus OX=9986 GN=CA3 PE=3 SV=2
119	1	Custom	tr|G1SZ14|G1SZ14_RABIT	114	0.08	36740	0.04	2	1	Proteasome subunit alpha type-3 OS=Oryctolagus cuniculus OX=9986 GN=PSMA3 PE=3 SV=2
168	1	Custom	tr|A0A5F9DNN9|A0A5F9DNN9_RABIT	34	0.08	36824	0.06	1	1	Chloride intracellular channel protein OS=Oryctolagus cuniculus OX=9986 GN=CLIC4 PE=3 SV=1
212	1	Custom	tr|G1SWI7|G1SWI7_RABIT	22	0.08	38869	0.06	1	1	Proteasome 20S subunit alpha 8 OS=Oryctolagus cuniculus OX=9986 GN=PSMA8 PE=3 SV=3
160	1	Custom	tr|A0A5F9DBZ8|A0A5F9DBZ8_RABIT	40	0.08	40286	0.05	4	1	Terminal uridylyl transferase 4 OS=Oryctolagus cuniculus OX=9986 GN=TUT4 PE=4 SV=1
204	1	Custom	tr|A0A5F9CHI3|A0A5F9CHI3_RABIT	24	0.08	40949	0.02	1	1	F-box protein 8 OS=Oryctolagus cuniculus OX=9986 GN=FBXO8 PE=4 SV=1
152	1	Custom	tr|A0A5F9CPK4|A0A5F9CPK4_RABIT	48	0.08	41022	0.04	1	1	Proteasome endopeptidase complex OS=Oryctolagus cuniculus OX=9986 GN=PSMB1 PE=4 SV=1
126	1	Custom	tr|G1SKS9|G1SKS9_RABIT	98	0.08	83193	0.07	3	2	Thioredoxin-disulfide reductase OS=Oryctolagus cuniculus OX=9986 GN=TXNRD1 PE=3 SV=3
211	1	Custom	tr|G1T671|G1T671_RABIT	22	0.08	41300	0.07	1	1	Acetyl-CoA acetyltransferase 2 OS=Oryctolagus cuniculus OX=9986 GN=ACAT2 PE=3 SV=3
76	1	Custom	tr|A0A5F9C0T5|A0A5F9C0T5_RABIT	481	0.07	84138	0.07	12	2	Actin-depolymerizing factor OS=Oryctolagus cuniculus OX=9986 GN=GSN PE=3 SV=1
199	1	Custom	tr|G1TG30|G1TG30_RABIT	25	0.07	42021	0.09	1	1	Creatine kinase OS=Oryctolagus cuniculus OX=9986 GN=CKM PE=3 SV=2
214	1	Custom	tr|A0A5F9CNT6|A0A5F9CNT6_RABIT	21	0.07	42383	0.02	1	1	Septin 12 OS=Oryctolagus cuniculus OX=9986 GN=SEPTIN12 PE=3 SV=1
54	1	Custom	tr|A0A5F9C1E9|A0A5F9C1E9_RABIT	996	0.07	43457	0.06	15	1	15-oxoprostaglandin 13-reductase OS=Oryctolagus cuniculus OX=9986 GN=PTGR1 PE=3 SV=1
110	1	Custom	tr|G1SM91|G1SM91_RABIT	156	0.07	43820	0.04	2	1	Fumarylacetoacetase OS=Oryctolagus cuniculus OX=9986 GN=FAH PE=3 SV=2
111	1	Custom	tr|A0A5F9DC04|A0A5F9DC04_RABIT	147	0.07	43840	0.06	3	1	Corticosteroid-binding globulin OS=Oryctolagus cuniculus OX=9986 GN=SERPINA6 PE=3 SV=1

180	1	Custom	tr|B7NZM0|B7NZM0_RABIT	29	0.07	44137	0.05	1	1	Apolipoprotein A-IV (Predicted) OS=Oryctolagus cuniculus OX=9986 GN=APOA4 PE=3 SV=1
195	1	Custom	tr|G1STT3|G1STT3_RABIT	26	0.07	45654	0.04	1	1	Serpin family B member 10 OS=Oryctolagus cuniculus OX=9986 GN=SERPINB10 PE=3 SV=1
151	1	Custom	tr|A0A5F9D039|A0A5F9D039_RABIT	50	0.07	46047	0.05	1	1	LY6/PLAUR domain containing 3 OS=Oryctolagus cuniculus OX=9986 GN=LYPD3 PE=4 SV=1
197	1	Custom	tr|G1SLB2|G1SLB2_RABIT	26	0.07	46597	0.02	2	1	Vacuole membrane protein 1 OS=Oryctolagus cuniculus OX=9986 GN=VMP1 PE=4 SV=3
178	1	Custom	tr|G1TLY8|G1TLY8_RABIT	29	0.07	47043	0.01	4	1	Nucleobindin 2 OS=Oryctolagus cuniculus OX=9986 GN=NUCB2 PE=3 SV=1
104	1	Custom	tr|G1T1P8|G1T1P8_RABIT	177	0.07	47644	0.02	22	1	UDP-N-acetylglucosamine pyrophosphorylase 1 OS=Oryctolagus cuniculus OX=9986 GN=UAP1 PE=4 SV=1
188	1	Custom	tr|A0A5F9DQN1|A0A5F9DQN1_RABIT	28	0.06	47989	0.01	1	1	Leukotriene A(4) hydrolase OS=Oryctolagus cuniculus OX=9986 GN=LTA4H PE=3 SV=1
102	1	Custom	tr|G1T4H3|G1T4H3_RABIT	184	0.06	48367	0.05	10	1	Serpin family G member 1 OS=Oryctolagus cuniculus OX=9986 PE=3 SV=3
120	1	Custom	tr|A0A5F9D5Z5|A0A5F9D5Z5_RABIT	113	0.06	48819	0.03	2	1	LIM domain binding 1 OS=Oryctolagus cuniculus OX=9986 GN=LDB1 PE=3 SV=2
127	1	Custom	tr|G1TYT3|G1TYT3_RABIT	97	0.06	98489	0.08	3	2	Methyltransferase-like protein OS=Oryctolagus cuniculus OX=9986 GN=METTL8 PE=3 SV=3
97	1	Custom	tr|A0A5F9DMU6|A0A5F9DMU6_RABIT	244	0.06	98697	0.03	11	1	PRP18 homolog OS=Oryctolagus cuniculus OX=9986 PE=3 SV=1
156	1	Custom	tr|A0A5F9D1H7|A0A5F9D1H7_RABIT	45	0.06	50873	0.04	2	1	Protein disulfide isomerase family A member 6 OS=Oryctolagus cuniculus OX=9986 GN=PDIA6 PE=3 SV=2
143	1	Custom	tr|A0A5F9DU85|A0A5F9DU85_RABIT	56	0.06	51850	0.05	1	1	Alpha-galactosidase OS=Oryctolagus cuniculus OX=9986 GN=NAGA PE=3 SV=1
206	1	Custom	tr|B7NZR4|B7NZR4_RABIT	24	0.06	52595	0.03	1	1	Cadherin-1 OS=Oryctolagus cuniculus OX=9986 GN=CDH1 PE=4 SV=3
208	1	Custom	tr|G1T6M0|G1T6M0_RABIT	23	0.06	52669	0.06	1	1	Inter-alpha-trypsin inhibitor heavy chain H3 OS=Oryctolagus cuniculus OX=9986 GN=ITIH3 PE=3 SV=1
148	1	Custom	tr|A0A5F9CDL7|A0A5F9CDL7_RABIT	51	0.06	53380	0.05	3	1	Dihydrolipoyl dehydrogenase OS=Oryctolagus cuniculus OX=9986 PE=3 SV=1
99	1	Custom	tr|A0A5F9C901|A0A5F9C901_RABIT	194	0.06	53876	0.02	9	1	Citrate synthase OS=Oryctolagus cuniculus OX=9986 GN=CS PE=3 SV=1

108	1	Custom	tr|A0A5F9CQN2|A0A5F9CQN2_RABIT	158	0.06	54372	0.02	3	1	55 kDa erythrocyte membrane protein OS=Oryctolagus cuniculus OX=9986 GN=MPP1 PE=4 SV=1
140	1	Custom Custom	tr|A0A5F9CXP2|A0A5F9CXP2_RABIT	66	0.05	56566	0.04	2	1	Endonuclease domain containing 1 OS=Oryctolagus cuniculus OX=9986 GN=ENDOD1 PE=4 SV=2
144	1	Custom	tr|A0A5F9CNG0|A0A5F9CNG0_RABIT	55	0.05	57314	0.02	1	1	Macrophage-capping protein OS=Oryctolagus cuniculus OX=9986 GN=CAPG PE=3 SV=1
163	1	Custom	tr|A0A5F9CFW1|A0A5F9CFW1_RABIT	39	0.05	57524	0.03	1	1	Bleomycin hydrolase OS=Oryctolagus cuniculus OX=9986 GN=BLMH PE=3 SV=1
162	1	Custom	tr|G1SCJ8|G1SCJ8_RABIT	39	0.05	57857	0.07	1	1	Peptidase D OS=Oryctolagus cuniculus OX=9986 GN=PEPD PE=4 SV=1
189	1	Custom	tr|A0A5F9CVJ5|A0A5F9CVJ5_RABIT	27	0.05	58400	0.02	2	1	Keratin 85 OS=Oryctolagus cuniculus OX=9986 GN=KRT85 PE=4 SV=1
158	1	Custom	tr|A0A5F9D9F5|A0A5F9D9F5_RABIT	45	0.05	58539	0.01	5	1	RCC1 and BTB domain containing protein 1 OS=Oryctolagus cuniculus OX=9986 GN=RCBTB1 PE=4 SV=1
171	1	Custom	tr|G1T466|G1T466_RABIT	33	0.05	119477	0.02	7	2	E1 ubiquitin-activating enzyme OS=Oryctolagus cuniculus OX=9986 GN=UBA6 PE=3 SV=1
198	1	Custom	tr|A0A5F9D0K7|A0A5F9D0K7_RABIT	25	0.05	59673	0.02	1	1	Uncharacterized protein OS=Oryctolagus cuniculus OX=9986 PE=4 SV=1
147	1	Custom	tr|G1T004|G1T004_RABIT	53	0.05	60133	0.06	1	1	Phosphoacetylglucosamine mutase OS=Oryctolagus cuniculus OX=9986 GN=PGM3 PE=3 SV=2
117	1	Custom	tr|A0A5F9CI66|A0A5F9CI66_RABIT	127	0.05	125209	0.04	3	2	Ceruloplasmin OS=Oryctolagus cuniculus OX=9986 GN=CP PE=3 SV=1
88	1	Custom	tr|A0A5F9C0P4|A0A5F9C0P4_RABIT	384	0.05	62283	0.02	6	1	Carboxylic ester hydrolase OS=Oryctolagus cuniculus OX=9986 PE=3 SV=1
218	1	Custom	tr|G1SZD9|G1SZD9_RABIT	20	0.05	62588	0.01	1	1	MFS domain-containing protein OS=Oryctolagus cuniculus OX=9986 PE=4 SV=2
100	1	Custom	tr|G1U974|G1U974_RABIT	194	0.05	62666	0.03	2	1	Dihydropyrimidinase-related protein 2 OS=Oryctolagus cuniculus OX=9986 GN=DPYSL2 PE=3 SV=1
203	1	Custom	tr|G1TTI1|G1TTI1_RABIT	24	0.05	63589	0.04	2	1	Uncharacterized protein OS=Oryctolagus cuniculus OX=9986 PE=4 SV=3
200	1	Custom	tr|A0A5F9CAT8|A0A5F9CAT8_RABIT	25	0.05	64434	0.03	1	1	Cystathionine beta-synthase OS=Oryctolagus cuniculus OX=9986 PE=3 SV=1
217	1	Custom	tr|G1TBI9|G1TBI9_RABIT	20	0.05	65148	0.02	1	1	Solute carrier family 17 member 6 OS=Oryctolagus cuniculus OX=9986 GN=SLC17A6 PE=4 SV=1

196	1	Custom	tr|A0A5F9CG66|A0A5F9CG66_RABIT	26	0.05	66092	0.01	1	1	E74 like ETS transcription factor 1 OS=Oryctolagus cuniculus OX=9986 GN=ELF1 PE=3 SV=1
125	1	Custom	tr|A0A5F9C396|A0A5F9C396_RABIT	100	0.05	67854	0.02	2	1	Phospholipase D family member 3 OS=Oryctolagus cuniculus OX=9986 GN=PLD3 PE=3 SV=1
134	1	Custom	tr|G1T3E2|G1T3E2_RABIT	80	0.05	68239	0.01	6	1	Transmembrane 9 superfamily member OS=Oryctolagus cuniculus OX=9986 GN=TM9SF3 PE=3 SV=2
137	1	Custom	tr|A0A5F9DE89|A0A5F9DE89_RABIT	70	0.04	70377	0.04	2	1	Afamin OS=Oryctolagus cuniculus OX=9986 GN=AFM PE=4 SV=1
153	1	Custom	tr|G1SH26|G1SH26_RABIT	47	0.04	71985	0.04	1	1	Uncharacterized protein OS=Oryctolagus cuniculus OX=9986 PE=4 SV=3
142	1	Custom	tr|A0A5F9D448|A0A5F9D448_RABIT	61	0.04	76614	0.01	2	1	IKs producing slow voltage-gated potassium channel subunit alpha KvLQT1 OS=Oryctolagus cuniculus OX=9986 GN=KCNQ1 PE=3 SV=1
191	1	Custom	tr|G1SKP6|G1SKP6_RABIT	27	0.04	76702	0.01	1	1	Leucine rich repeat, Ig-like and transmembrane domains 3 OS=Oryctolagus cuniculus OX=9986 GN=LRIT3 PE=4 SV=3
109	1	Custom	tr|A0A5F9CTS6|A0A5F9CTS6_RABIT	157	0.04	79476	0.04	4	1	Uncharacterized protein OS=Oryctolagus cuniculus OX=9986 PE=4 SV=1
124	1	Custom	tr|A0A5F9CH47|A0A5F9CH47_RABIT	103	0.04	81577	0.01	12	1	Catenin beta 1 OS=Oryctolagus cuniculus OX=9986 GN=CTNNB1 PE=3 SV=1
123	1	Custom	tr|A0A5F9DNV0|A0A5F9DNV0_RABIT	103	0.03	174711	0.03	3	2	C4a anaphylatoxin OS=Oryctolagus cuniculus OX=9986 PE=4 SV=1
202	1	Custom	tr|A0A5F9C5U6|A0A5F9C5U6_RABIT	24	0.03	91896	0.01	1	1	Zinc finger FYVE-type containing 1 OS=Oryctolagus cuniculus OX=9986 GN=ZFYVE1 PE=4 SV=1
106	1	Custom	tr|G1TEV3|G1TEV3_RABIT	170	0.03	92889	0.01	9	1	PiggyBac transposable element derived 1 OS=Oryctolagus cuniculus OX=9986 GN=PGBD1 PE=4 SV=1
103	1	contaminants	Q2UVX4	181	0.03	188789	0.01	5	2	SWISS-PROT:Q2UVX4 (Bos taurus) Complement C3 precursor
205	1	Custom	tr|A0A5F9D2E7|A0A5F9D2E7_RABIT	24	0.03	94456	0.01	1	1	ADAM metallopeptidase domain 23 OS=Oryctolagus cuniculus OX=9986 GN=ADAM23 PE=4 SV=1
167	1	Custom	tr|A0A5F9C3H6|A0A5F9C3H6_RABIT	35	0.03	96357	0.03	1	1	Inter-alpha-trypsin inhibitor heavy chain 2 OS=Oryctolagus cuniculus OX=9986 GN=ITIH2 PE=3 SV=1
122	1	contaminants	Q3T052	104	0.03	101620	0.01	10	1	TREMBL:Q3T052;Q5EA67 (Bos taurus) Inter-alpha (Globulin) inhibitor H4

182	1	Custom	tr|A0A5F9C0T6|A0A5F9C0T6_RABIT	29	0.03	101578	0.01	2	1	VWFA domain-containing protein OS=Oryctolagus cuniculus OX=9986 PE=3 SV=1
210	1	Custom	tr|A0A5F9CS70|A0A5F9CS70_RABIT	22	0.03	108026	0.02	1	1	Centrosomal protein 112 OS=Oryctolagus cuniculus OX=9986 GN=CEP112 PE=4 SV=1
201	1	Custom	tr|A0A5F9C902|A0A5F9C902_RABIT	25	0.03	108296	0.02	2	1	Uncharacterized protein OS=Oryctolagus cuniculus OX=9986 PE=4 SV=1
187	1	Custom	tr|G1TLT6|G1TLT6_RABIT	28	0.03	109633	0.01	2	1	Ankyrin repeat domain 35 OS=Oryctolagus cuniculus OX=9986 GN=ANKRD35 PE=4 SV=2
177	1	Custom	tr|A0A5F9CFU7|A0A5F9CFU7_RABIT	30	0.03	113678	0.01	1	1	C2 calcium dependent domain containing 5 OS=Oryctolagus cuniculus OX=9986 GN=C2CD5 PE=4 SV=1
219	1	Custom	tr|A0A5F9C4T6|A0A5F9C4T6_RABIT	20	0.03	117795	0.01	1	1	PRP6 homolog OS=Oryctolagus cuniculus OX=9986 GN=PRPF6 PE=4 SV=1
170	1	Custom	tr|G1SV13|G1SV13_RABIT	33	0.03	118710	0.02	1	1	Ubiquitin-activating enzyme E1 OS=Oryctolagus cuniculus OX=9986 GN=UBA1 PE=3 SV=1
194	1	Custom	tr|G1SXP0|G1SXP0_RABIT	26	0.02	125399	0.01	2	1	Guanylate cyclase OS=Oryctolagus cuniculus OX=9986 GN=GUCY2F PE=3 SV=2
183	1	Custom	tr|A0A5F9CHG4|A0A5F9CHG4_RABIT	28	0.02	126858	0.01	1	1	Dynactin subunit 1 OS=Oryctolagus cuniculus OX=9986 GN=DCTN1 PE=4 SV=1
184	1	Custom	tr|A0A5F9CFK2|A0A5F9CFK2_RABIT	28	0.02	133231	0.01	1	1	Formin 1 OS=Oryctolagus cuniculus OX=9986 GN=FMN1 PE=3 SV=1
175	1	Custom	tr|A0A5F9CHJ8|A0A5F9CHJ8_RABIT	31	0.02	134271	0.01	2	1	Reverse transcriptase domain-containing protein OS=Oryctolagus cuniculus OX=9986 PE=4 SV=1
174	1	Custom	tr|A0A5F9D0L4|A0A5F9D0L4_RABIT	31	0.02	134836	0.01	1	1	Tyrosine-protein phosphatase non-receptor type OS=Oryctolagus cuniculus OX=9986 GN=PTPN21 PE=3 SV=1
213	1	Custom	tr|G1SIP7|G1SIP7_RABIT	21	0.02	137476	0.01	1	1	Cyclin G associated kinase OS=Oryctolagus cuniculus OX=9986 GN=GAK PE=4 SV=3
169	1	Custom	tr|A0A5F9CAH1|A0A5F9CAH1_RABIT	33	0.02	161611	0.01	5	1	ATP binding cassette subfamily C member 5 OS=Oryctolagus cuniculus OX=9986 GN=ABCC5 PE=4 SV=1
179	1	Custom	tr|A0A5F9CAI2|A0A5F9CAI2_RABIT	29	0.02	162482	0.01	2	1	Adhesion G protein-coupled receptor L3 OS=Oryctolagus cuniculus OX=9986 GN=ADGRL3 PE=4 SV=1
164	1	Custom	tr|G1SSU6|G1SSU6_RABIT	39	0.02	164193	0	2	1	Contactin associated protein 1 OS=Oryctolagus cuniculus OX=9986 GN=CNTNAP1 PE=3 SV=2

161	1	Custom	tr|A0A5F9DHA0|A0A5F9DHA0_RABIT	40	0.02	169630	0	3	1	URB2 ribosome biogenesis homolog OS=Oryctolagus cuniculus OX=9986 GN=URB2 PE=4 SV=1
193	1	Custom	tr|A0A5F9C8M0|A0A5F9C8M0_RABIT	26	0.01	235323	0.01	1	1	HEAT repeat-containing protein 1 OS=Oryctolagus cuniculus OX=9986 GN=HEATR1 PE=3 SV=1
159	1	Custom	tr|G1SE95|G1SE95_RABIT	40	0.01	279626	0.01	3	1	Uncharacterized protein OS=Oryctolagus cuniculus OX=9986 PE=3 SV=2
190	1	Custom	tr|G1SSJ4|G1SSJ4_RABIT	27	0.01	403121	0	1	1	CUB and Sushi multiple domains 2 OS=Oryctolagus cuniculus OX=9986 GN=CSMD2 PE=4 SV=2
192	1	Custom	tr|G1T093|G1T093_RABIT	26	0.01	525889	0	2	1	Sacsin molecular chaperone OS=Oryctolagus cuniculus OX=9986 GN=SACS PE=4 SV=1

Database 1: contaminants 20160129 (249 sequences; 128730 residues); database 2: Custom (41459 sequences; 24801325 residues); type of search: MS/MS Ion Search; enzyme: trypsin; fixed modifications: Carbamidomethyl (C); variable modifications: deamidated (NQ), oxidation (M); mass values: monoisotopic; protein mass:unrestricted; peptide mass tolerance: 3 ppm; fragment mass tolerance: 0.6 Da; max missed cleavages: 2; instrument type: ESI-TRAP; number of queries: 28447; significance threshold: $p < 0.05$; max. number of families: AUTO; ions score or expect cut-off: 20; preferred taxonomy: all entries.

Table S2. Proteins identified by LC-MS/MS with Mascot for ULu.

Family	Member	database	Accession	Score	emPAI	Mass	Sequence coverage	Num. of significant matches	Num. of significant unique sequences	description
1	1	Custom	sp|P02057|HBB_RABIT	45846	107.44	16179	0.9	1281	8	Hemoglobin subunit beta-1/2 OS=Oryctolagus cuniculus OX=9986 GN=HBB1 PE=1 SV=2
1	2	Custom	tr|A0A5F9D756|A0A5F9D756_RABIT	32172	69.47	16547	0.88	971	8	Hemoglobin subunit gamma OS=Oryctolagus cuniculus OX=9986 PE=3 SV=1
2	1	Custom	tr|G1U9S2|G1U9S2_RABIT	40704	9.3	70916	0.76	1201	42	Albumin OS=Oryctolagus cuniculus OX=9986 GN=ALB PE=1 SV=1
2	2	contaminants	P02768-1	4219	0.39	71317	0.1	192	1	SWISS-PROT:P02768-1 Tax_Id=9606 Gene_Symbol=ALB Isoform 1 of Serum albumin precursor
2	3	contaminants	P02769	2838	0.57	71244	0.19	89	3	SWISS-PROT:P02769 (Bos taurus) Bovine serum albumin precursor
3	1	Custom	tr|G1STF7|G1STF7_RABIT	17688	3.69	78916	0.55	506	39	Beta-1 metal-binding globulin OS=Oryctolagus cuniculus OX=9986 PE=3 SV=1
4	1	Custom	tr|G1T9M9|G1T9M9_RABIT	13589	2.14	71082	0.43	325	22	Heat shock protein family A (Hsp70) member 8 OS=Oryctolagus cuniculus OX=9986 PE=3 SV=2
4	2	Custom	tr|A0A5F9C804|A0A5F9C804_RABIT	6579	0.91	71475	0.34	138	14	78 kDa glucose-regulated protein OS=Oryctolagus cuniculus OX=9986 GN=HSPA5 PE=3 SV=1

5	1	Custom	tr|A0A5F9C3B7|A0A5F9C3B7_RABIT	12361	1.1	126137	0.37	303	32	Metavinculin OS=Oryctolagus cuniculus OX=9986 GN=VCL PE=3 SV=1
6	1	Custom	sp|Q8MI17|AL1A1_RABIT	12223	3.86	54933	0.7	334	24	Retinal dehydrogenase 1 OS=Oryctolagus cuniculus OX=9986 GN=ALDH1A1 PE=1 SV=1
6	2	Custom	tr|A0A5F9DBD2|A0A5F9DBD2_RABIT	2040	0.67	73710	0.25	67	10	Aldedh domain-containing protein OS=Oryctolagus cuniculus OX=9986 PE=3 SV=1
7	1	Custom	tr|G1TET2|G1TET2_RABIT	12024	3.28	71937	0.69	329	29	Lymphocyte cytosolic protein 1 OS=Oryctolagus cuniculus OX=9986 GN=LCP1 PE=4 SV=2
7	2	Custom	tr|G1TRY5|G1TRY5_RABIT	3414	0.44	71307	0.14	92	4	Plastin 3 OS=Oryctolagus cuniculus OX=9986 GN=PLS3 PE=4 SV=2
8	1	Custom	sp|P01948|HBA_RABIT	11527	20.13	15636	0.89	419	13	Hemoglobin subunit alpha-1/2 OS=Oryctolagus cuniculus OX=9986 PE=1 SV=2
9	1	Custom	tr|G1SWS9|G1SWS9_RABIT	7697	2.84	53679	0.48	203	22	Vimentin OS=Oryctolagus cuniculus OX=9986 GN=VIM PE=3 SV=1
9	2	Custom	tr|G1SHZ4|G1SHZ4_RABIT	3720	1.63	41748	0.39	98	12	Keratin 7 OS=Oryctolagus cuniculus OX=9986 GN=KRT7 PE=3 SV=3
9	3	Custom	tr|G1SS18|G1SS18_RABIT	3611	1.11	54172	0.29	110	10	IF rod domain-containing protein OS=Oryctolagus cuniculus OX=9986 PE=3 SV=2
9	4	contaminants	P04264	3205	0.48	66149	0.17	66	7	SWISS-PROT:P04264 Tax_Id=9606 Gene_Symbol=KRT1 Keratin, type II cytoskeletal 1
9	5	contaminants	P35908	1028	0.19	66110	0.07	28	2	SWISS-PROT:P35908 Tax_Id=9606 Gene_Symbol=KRT2 Keratin, type II cytoskeletal 2 epidermal
9	6	contaminants	Q8BGZ7	857	0.27	59932	0.09	37	1	TREMBL:Q8BGZ7;Q99MH7 Tax_Id=10090 Gene_Symbol=Krt75 10 days neonate skin cDNA, RIKEN full-length enriched library, clone:4732475I03 product:CYTOKERATIN homolog
9	7	Custom	tr|G1U9G5|G1U9G5_RABIT	152	0.11	55685	0.04	6	1	Keratin 78 OS=Oryctolagus cuniculus OX=9986 GN=KRT78 PE=3 SV=2
10	1	Custom	tr|A0A5F9DVM5|A0A5F9DVM5_RABIT	7503	2.1	58969	0.51	241	2	SEC14 like lipid binding 3 OS=Oryctolagus cuniculus OX=9986 GN=SEC14L3 PE=4 SV=1
10	2	Custom	tr|G1T6Z0|G1T6Z0_RABIT	7478	2.37	54870	0.54	257	2	SEC14 like lipid binding 3 OS=Oryctolagus cuniculus OX=9986 GN=SEC14L3 PE=4 SV=2
11	1	Custom	tr|A0A5F9D287|A0A5F9D287_RABIT	7441	2.27	48814	0.58	176	17	2-phospho-D-glycerate hydro-lyase OS=Oryctolagus cuniculus OX=9986 PE=3 SV=1
12	1	Custom	tr|G1TFX2|G1TFX2_RABIT	7303	1.48	50843	0.42	189	4	SERPIN domain-containing protein OS=Oryctolagus cuniculus OX=9986 GN=SERPINA1 PE=3 SV=2
12	2	Custom	sp|P23035|A1AF_RABIT	6098	1.41	46010	0.38	159	3	Alpha-1-antiproteinase F OS=Oryctolagus cuniculus OX=9986 PE=1 SV=1
13	1	Custom	tr|G1THZ6|G1THZ6_RABIT	7171	3.31	37369	0.7	226	3	Immunoglobulin heavy constant mu OS=Oryctolagus cuniculus OX=9986 GN=IGHM PE=4 SV=3

13	2	Custom	sp|P01870|IGHG_RABIT	5554	2.59	35952	0.63	199	1	Ig gamma chain C region OS=Oryctolagus cuniculus OX=9986 PE=1 SV=1
14	1	Custom	tr|A0A5F9C1L6|A0A5F9C1L6_RABIT	6619	0.66	97463	0.25	173	9	Actinin alpha 1 OS=Oryctolagus cuniculus OX=9986 GN=ACTN1 PE=4 SV=1
14	2	Custom	tr|A0A5F9D4S1|A0A5F9D4S1_RABIT	4704	0.89	73305	0.35	137	8	Actinin alpha 4 OS=Oryctolagus cuniculus OX=9986 GN=ACTN4 PE=4 SV=1
15	1	Custom	tr|A0A5F9D1J7|A0A5F9D1J7_RABIT	6613	2.45	56068	0.59	178	22	Selenium binding protein 1 OS=Oryctolagus cuniculus OX=9986 GN=SELENBP1 PE=3 SV=1
16	1	Custom	tr|A0A5F9CVU0|A0A5F9CVU0_RABIT	6576	2.16	47748	0.37	167	14	Tyrosine 3-monooxygenase/tryptophan 5-monooxygenase activation protein epsilon OS=Oryctolagus cuniculus OX=9986 GN=YWHAE PE=3 SV=1
16	2	Custom	tr|A0A5F9C4B7|A0A5F9C4B7_RABIT	6096	2.33	28572	0.48	125	9	Tyrosine 3-monooxygenase/tryptophan 5-monooxygenase activation protein zeta OS=Oryctolagus cuniculus OX=9986 GN=YWHAZ PE=3 SV=1
16	3	Custom	tr|G1TZP0|G1TZP0_RABIT	3317	1.48	28412	0.39	83	4	Tyrosine 3-monooxygenase/tryptophan 5-monooxygenase activation protein gamma OS=Oryctolagus cuniculus OX=9986 GN=YWHAG PE=3 SV=1
16	4	Custom	tr|G1SZD6|G1SZD6_RABIT	2682	1.04	28044	0.26	74	3	14-3-3 protein theta OS=Oryctolagus cuniculus OX=9986 GN=YWHAQ PE=3 SV=1
16	5	Custom	tr|B7NZM8|B7NZM8_RABIT	2608	1.25	28351	0.31	75	4	Tyrosine 3-monooxygenase/tryptophan 5-monooxygenase activation protein eta OS=Oryctolagus cuniculus OX=9986 GN=YWHAH PE=3 SV=1
16	6	Custom	tr|A0A5F9CN85|A0A5F9CN85_RABIT	2225	0.63	41097	0.16	70	2	Tyrosine 3-monooxygenase/tryptophan 5-monooxygenase activation protein beta OS=Oryctolagus cuniculus OX=9986 GN=YWHAB PE=3 SV=1
17	1	Custom	tr|A0A5F9C312|A0A5F9C312_RABIT	5818	3.58	33979	0.71	159	1	Aldo_ket_red domain-containing protein OS=Oryctolagus cuniculus OX=9986 PE=3 SV=1
17	2	Custom	tr|A0A5F9CBR3|A0A5F9CBR3_RABIT	5682	3.31	35454	0.7	157	1	Aldo_ket_red domain-containing protein OS=Oryctolagus cuniculus OX=9986 PE=3 SV=1
18	1	Custom	sp|P12337|EST1_RABIT	5784	1	62594	0.35	154	1	Liver carboxylesterase 1 OS=Oryctolagus cuniculus OX=9986 PE=1 SV=3
18	2	Custom	tr|A0A5F9CDQ0|A0A5F9CDQ0_RABIT	5548	1.07	59653	0.37	151	1	Carboxylic ester hydrolase OS=Oryctolagus cuniculus OX=9986 PE=3 SV=1
19	1	Custom	sp|Q9TTC6|PPIA_RABIT	5592	4.57	18054	0.72	147	9	Peptidyl-prolyl cis-trans isomerase A OS=Oryctolagus cuniculus OX=9986 GN=PPIA PE=2 SV=3
20	1	Custom	sp|P00939|TPI	5468	2.57	27025	0.69	116	12	Triosephosphate isomerase OS=Oryctolagus cuniculus

			S_RABIT							OX=9986 GN=TPI1 PE=1 SV=3
21	1	Custom	sp|P01832|PIGR_RABIT	5018	0.41	84975	0.19	95	10	Polymeric immunoglobulin receptor OS=Oryctolagus cuniculus OX=9986 GN=PIGR PE=1 SV=1
22	1	contaminants	P60712	4963	1.28	42052	0.42	126	7	SWISS-PROT:P60712 (Bos taurus) Actin, cytoplasmic 1
22	2	Custom	tr|G1SDT0|G1SDT0_RABIT	2665	0.51	41913	0.21	68	1	Actin beta like 2 OS=Oryctolagus cuniculus OX=9986 GN=ACTBL2 PE=3 SV=2
23	1	Custom	tr|A0A5F9C503|A0A5F9C503_RABIT	4941	0.92	84618	0.3	145	12	Heat shock protein 90 alpha family class A member 1 OS=Oryctolagus cuniculus OX=9986 PE=3 SV=1
23	2	Custom	tr|G1TBC1|G1TBC1_RABIT	3378	0.55	92618	0.22	112	13	Endoplasmin OS=Oryctolagus cuniculus OX=9986 GN=HSP90B1 PE=3 SV=2
23	3	Custom	sp|P30947|HS90B_RABIT	2101	0.46	83757	0.17	58	3	Heat shock protein HSP 90-beta OS=Oryctolagus cuniculus OX=9986 GN=HSP90AB1 PE=1 SV=2
24	1	Custom	tr|A0A5F9CAH6|A0A5F9CAH6_RABIT	4835	0.54	162896	0.26	148	23	Alpha-2-macroglobulin OS=Oryctolagus cuniculus OX=9986 GN=A2M PE=3 SV=1
25	1	Custom	tr|G1T0R9|G1T0R9_RABIT	4558	3.75	25652	0.63	109	2	Glutathione S-transferase OS=Oryctolagus cuniculus OX=9986 GN=GSTM3 PE=3 SV=1
25	2	Custom	tr|G1TY06|G1TY06_RABIT	2971	2.04	25690	0.44	70	1	Glutathione S-transferase OS=Oryctolagus cuniculus OX=9986 PE=3 SV=3
25	3	Custom	tr|A0A5F9DRY0|A0A5F9DRY0_RABIT	2473	1.24	21098	0.28	56	1	Uncharacterized protein OS=Oryctolagus cuniculus OX=9986 GN=GSTM2 PE=4 SV=1
25	4	Custom	tr|A0A5F9DDG6|A0A5F9DDG6_RABIT	2261	1.44	25652	0.42	57	1	Glutathione S-transferase OS=Oryctolagus cuniculus OX=9986 GN=GSTM2 PE=3 SV=1
25	5	Custom	tr|G1SXP3|G1SXP3_RABIT	1139	1.84	10559	0.53	35	2	GST C-terminal domain-containing protein OS=Oryctolagus cuniculus OX=9986 PE=4 SV=2
25	6	Custom	tr|G1SXQ0|G1SXQ0_RABIT	1098	0.41	33150	0.17	30	4	Glutathione S-transferase OS=Oryctolagus cuniculus OX=9986 GN=GSTM3 PE=3 SV=2
26	1	Custom	tr|G1T2C4|G1T2C4_RABIT	4468	4.12	22685	0.57	121	10	Transgelin OS=Oryctolagus cuniculus OX=9986 PE=3 SV=2
27	1	Custom	sp|O97972|INMT_RABIT	4392	2.2	29507	0.45	140	10	Indolethylamine N-methyltransferase OS=Oryctolagus cuniculus OX=9986 GN=INMT PE=1 SV=1
28	1	Custom	sp|P21195|PDIA1_RABIT	4230	1.14	57172	0.35	118	14	Protein disulfide-isomerase OS=Oryctolagus cuniculus OX=9986 GN=P4HB PE=2 SV=1
29	1	Custom	tr|A0A5F9CQ70|A0A5F9CQ70_RABIT	4157	3.59	24329	0.64	149	13	Thioredoxin domain-containing protein OS=Oryctolagus cuniculus OX=9986 PE=3 SV=1
30	1	Custom	sp|P00919|CAH2_RABIT	3849	2.19	29596	0.49	109	11	Carbonic anhydrase 2 OS=Oryctolagus cuniculus OX=9986 GN=CA2 PE=1 SV=3
31	1	Custom	tr|G1SER8|G1SER8_RABIT	3837	5.93	11404	0.69	76	2	Profilin OS=Oryctolagus cuniculus OX=9986 GN=PFN1 PE=3 SV=2

31	2	contaminants	P02584	2197	2.02	15219	0.41	53	1	SWISS-PROT:P02584 (Bos taurus) Profilin-1
32	1	Custom	sp|P15253|CALR_RABIT	3818	1.6	48416	0.58	179	16	Calreticulin OS=Oryctolagus cuniculus OX=9986 GN=CALR PE=1 SV=1
33	1	Custom	tr|G1SQG5|G1SQG5_RABIT	3796	1.37	36643	0.45	98	11	Malate dehydrogenase OS=Oryctolagus cuniculus OX=9986 GN=MDH1 PE=3 SV=1
34	1	Custom	tr|G1TKE3|G1TKE3_RABIT	3746	2.04	20402	0.36	73	5	Transgelin OS=Oryctolagus cuniculus OX=9986 GN=TAGLN2 PE=3 SV=2
35	1	Custom	tr|A0A5F9CRZ7|A0A5F9CRZ7_RABIT	3621	1.53	49870	0.39	101	14	Keratin 15 OS=Oryctolagus cuniculus OX=9986 GN=KRT15 PE=3 SV=1
35	2	Custom	tr|G1TMP1|G1TMP1_RABIT	2369	1.09	46988	0.33	70	10	IF rod domain-containing protein OS=Oryctolagus cuniculus OX=9986 PE=4 SV=2
35	3	contaminants	P13645	1605	0.55	59703	0.2	36	8	SWISS-PROT:P13645 Tax_Id=9606 Gene_Symbol=KRT10 Keratin, type I cytoskeletal 10
35	4	contaminants	P35527	975	0.26	62320	0.13	25	4	SWISS-PROT:P35527 Tax_Id=9606 Gene_Symbol=KRT9 Keratin, type I cytoskeletal 9
36	1	Custom	tr|A0A5F9DEF9|A0A5F9DEF9_RABIT	3539	1.96	23630	0.5	97	7	GST class-pi OS=Oryctolagus cuniculus OX=9986 PE=3 SV=1
37	1	Custom	tr|A0A5F9CNW6|A0A5F9CNW6_RABIT	3147	1.09	59107	0.37	75	14	Glucose-6-phosphate isomerase OS=Oryctolagus cuniculus OX=9986 GN=GPI PE=3 SV=1
38	1	Custom	tr|G1T823|G1T823_RABIT	3144	1.16	26021	0.31	81	3	ADF-H domain-containing protein OS=Oryctolagus cuniculus OX=9986 PE=4 SV=3
38	2	Custom	tr|A0A5F9DL18|A0A5F9DL18_RABIT	2660	0.76	25330	0.28	49	1	ADF-H domain-containing protein OS=Oryctolagus cuniculus OX=9986 PE=4 SV=1
39	1	Custom	tr|G1T7R1|G1T7R1_RABIT	3081	2.12	14806	0.41	78	5	Fatty acid binding protein 3 OS=Oryctolagus cuniculus OX=9986 GN=FABP3 PE=3 SV=1
40	1	Custom	tr|G1T804|G1T804_RABIT	2959	0.65	34578	0.31	44	6	Uncharacterized protein OS=Oryctolagus cuniculus OX=9986 PE=4 SV=2
41	1	Custom	sp|P07452|CAH1_RABIT	2846	1.71	25737	0.57	76	8	Carbonic anhydrase 1 (Fragment) OS=Oryctolagus cuniculus OX=9986 GN=CA1 PE=2 SV=1
41	2	Custom	tr|A0A5F9DUH1|A0A5F9DUH1_RABIT	475	0.76	9674	0.31	15	1	Carbonate dehydratase I OS=Oryctolagus cuniculus OX=9986 PE=3 SV=1
42	1	Custom	tr|G1SQ02|G1SQ02_RABIT	2794	3.62	22366	0.69	93	11	Peroxiredoxin-1 OS=Oryctolagus cuniculus OX=9986 PE=3 SV=1
43	1	Custom	tr|G1SP97|G1SP97_RABIT	2539	0.35	38736	0.16	53	4	Lumican OS=Oryctolagus cuniculus OX=9986 GN=LUM PE=3 SV=1
44	1	Custom	tr|A0A5S8H274|A0A5S8H274_RABIT	2530	2.53	27249	0.53	94	12	Glutathione S-transferase Yc OS=Oryctolagus cuniculus OX=9986 GN=GSTA5 PE=3 SV=1

45	1	Custom	tr|U3KMR2|U3KMR2_RABIT	2448	0.6	36689	0.26	62	6	Alpha-2-HS-glycoprotein OS=Oryctolagus cuniculus OX=9986 GN=AHSG PE=4 SV=1
46	1	Custom	tr|G1U974|G1U974_RABIT	2423	0.66	62666	0.28	51	10	Dihydropyrimidinase-related protein 2 OS=Oryctolagus cuniculus OX=9986 GN=DPYSL2 PE=3 SV=1
47	1	Custom	tr|A0A5F9CII3|A0A5F9CII3_RABIT	2317	1.44	28849	0.25	53	2	Chromosome 1 open reading frame 43 OS=Oryctolagus cuniculus OX=9986 GN=C1orf43 PE=3 SV=1
47	2	Custom	tr|A0A5F9DVE1|A0A5F9DVE1_RABIT	1390	0.85	37548	0.16	49	5	Tropomyosin 4 OS=Oryctolagus cuniculus OX=9986 GN=TPM4 PE=3 SV=1
47	3	Custom	tr|A0A5F9DP16|A0A5F9DP16_RABIT	1370	1.18	33129	0.24	49	2	Chromosome 1 open reading frame 43 OS=Oryctolagus cuniculus OX=9986 GN=C1orf43 PE=3 SV=1
48	1	Custom	tr|A0A5F9CAX1|A0A5F9CAX1_RABIT	2333	0.83	28435	0.35	64	6	Biliverdin reductase B OS=Oryctolagus cuniculus OX=9986 GN=BLVRB PE=4 SV=1
49	1	Custom	tr|A0A5F9D486|A0A5F9D486_RABIT	2332	0.7	70926	0.35	72	13	Alpha fetoprotein OS=Oryctolagus cuniculus OX=9986 GN=AFP PE=4 SV=1
50	1	Custom	tr|A0A5F9DJP2|A0A5F9DJP2_RABIT	2215	0.83	57595	0.3	66	11	Catalase OS=Oryctolagus cuniculus OX=9986 GN=CAT PE=3 SV=1
51	1	Custom	tr|G1TV43|G1TV43_RABIT	2169	1	41753	0.36	60	10	Aldo-keto reductase family 1 member B10 OS=Oryctolagus cuniculus OX=9986 GN=AKR1B10 PE=3 SV=2
52	1	Custom	tr|A0A5F9CCJ1|A0A5F9CCJ1_RABIT	1992	0.26	63725	0.1	53	5	Gc-globulin OS=Oryctolagus cuniculus OX=9986 GN=GC PE=4 SV=1
53	1	Custom	tr|G1U7U3|G1U7U3_RABIT	1917	1.66	17315	0.41	74	1	Nucleoside diphosphate kinase OS=Oryctolagus cuniculus OX=9986 GN=NME1 PE=3 SV=2
53	2	Custom	tr|U3KNZ4|U3KNZ4_RABIT	1329	1	24747	0.31	61	1	Nucleoside diphosphate kinase OS=Oryctolagus cuniculus OX=9986 PE=3 SV=1
54	1	Custom	tr|A0A5F9C6Q3|A0A5F9C6Q3_RABIT	1855	2.41	13607	0.73	63	6	Transthyretin OS=Oryctolagus cuniculus OX=9986 GN=TTR PE=3 SV=1
55	1	Custom	tr|G1SN21|G1SN21_RABIT	1837	0.7	32277	0.29	38	6	Purine nucleoside phosphorylase OS=Oryctolagus cuniculus OX=9986 GN=PNP PE=3 SV=2
56	1	Custom	sp|P24480|S10AB_RABIT	1820	1.61	11536	0.33	42	3	Protein S100-A11 OS=Oryctolagus cuniculus OX=9986 GN=S100A11 PE=1 SV=2
57	1	Custom	tr|G1SNS5|G1SNS5_RABIT	1745	0.49	50916	0.19	54	2	Rab GDP dissociation inhibitor OS=Oryctolagus cuniculus OX=9986 PE=3 SV=2
57	2	Custom	tr|G1TE41|G1TE41_RABIT	1446	0.52	48611	0.23	45	2	Rab GDP dissociation inhibitor OS=Oryctolagus cuniculus OX=9986 GN=GDI1 PE=3 SV=1
58	1	Custom	sp|P01840|KA	1711	1.68	11264	0.63	41	3	Ig kappa-b4 chain C region OS=Oryctolagus cuniculus

			C4_RABIT							OX=9986 PE=1 SV=1
59	1	contaminants	P00761	1622	1.48	25078	0.25	80	4	SWISS-PROT:P00761|TRYP_PIG Trypsin - Sus scrofa (Pig).
60	1	Custom	tr|A0A5F9C0T5|A0A5F9C0T5_RABIT	1605	0.27	84138	0.14	33	7	Actin-depolymerizing factor OS=Oryctolagus cuniculus OX=9986 GN=GSN PE=3 SV=1
61	1	Custom	sp|P62160|CALM_RABIT	1585	0.65	16827	0.27	38	2	Calmodulin OS=Oryctolagus cuniculus OX=9986 GN=CALM PE=1 SV=2
62	1	Custom	tr|G1T7Z0|G1T7Z0_RABIT	1584	0.72	53445	0.22	51	10	6-phosphogluconate dehydrogenase, decarboxylating OS=Oryctolagus cuniculus OX=9986 GN=PGD PE=3 SV=3
63	1	Custom	tr|A0A5F9D5U7|A0A5F9D5U7_RABIT	1530	0.56	45609	0.21	52	7	Fructose-bisphosphate aldolase OS=Oryctolagus cuniculus OX=9986 PE=3 SV=1
64	1	Custom	sp|P23108|IGJ_RABIT	1522	1.02	16003	0.31	49	4	Immunoglobulin J chain OS=Oryctolagus cuniculus OX=9986 GN=JCHAIN PE=1 SV=1
65	1	Custom	tr|G1SE95|G1SE95_RABIT	1496	0.13	279626	0.08	41	12	Uncharacterized protein OS=Oryctolagus cuniculus OX=9986 PE=3 SV=2
66	1	Custom	tr|G1T6L5|G1T6L5_RABIT	1480	0.47	22255	0.23	23	3	SH3 domain binding glutamate rich protein like OS=Oryctolagus cuniculus OX=9986 GN=SH3BGRL PE=3 SV=2
67	1	Custom	tr|G1SV13|G1SV13_RABIT	1436	0.16	118710	0.1	33	6	Ubiquitin-activating enzyme E1 OS=Oryctolagus cuniculus OX=9986 GN=UBA1 PE=3 SV=1
68	1	Custom	tr|A0A5F9C0P4|A0A5F9C0P4_RABIT	1412	0.2	62283	0.11	28	4	Carboxylic ester hydrolase OS=Oryctolagus cuniculus OX=9986 PE=3 SV=1
69	1	Custom	tr|A0A5F9CFW1|A0A5F9CFW1_RABIT	1407	0.42	57524	0.21	35	7	Leukotriene A(4) hydrolase OS=Oryctolagus cuniculus OX=9986 GN=LTA4H PE=3 SV=1
70	1	Custom	tr|G1SV22|G1SV22_RABIT	1365	0.5	21110	0.33	18	3	Phosphatidylethanolamine-binding protein 1 OS=Oryctolagus cuniculus OX=9986 GN=PEBP1 PE=3 SV=2
71	1	Custom	tr|G1TVS4|G1TVS4_RABIT	1252	0.74	52317	0.26	77	10	Hemopexin OS=Oryctolagus cuniculus OX=9986 GN=TRIM3 PE=3 SV=2
72	1	Custom	tr|A0A5F9CAH3|A0A5F9CAH3_RABIT	1156	0.1	186643	0.08	20	6	IQ motif containing GTPase activating protein 1 OS=Oryctolagus cuniculus OX=9986 GN=IQGAP1 PE=4 SV=1
73	1	Custom	tr|G1SZV5|G1SZV5_RABIT	1121	0.45	30615	0.18	30	4	Apolipoprotein A-I OS=Oryctolagus cuniculus OX=9986 GN=APOA1 PE=3 SV=1
74	1	Custom	tr|A0A5F9CD26|A0A5F9CD26_RABIT	1114	0.34	39212	0.17	35	4	Haptoglobin OS=Oryctolagus cuniculus OX=9986 PE=3 SV=1
75	1	Custom	tr|G1SQ01|G1SQ01_RABIT	1095	0.36	47494	0.16	29	5	Aldo-keto reductase family 1 member A1 OS=Oryctolagus cuniculus OX=9986 GN=AKR1A1 PE=3 SV=2
76	1	Custom	tr|G1SPF5|G1S	1017	1.62	14574	0.54	47	3	Phosphoglycerate mutase 1 OS=Oryctolagus cuniculus

			PF5_RABIT							OX=9986 GN=PGAM1 PE=4 SV=2
76	2	Custom	tr|G1U7S4|G1U7S4_RABIT	152	0.22	28820	0.08	17	1	Phosphoglycerate mutase OS=Oryctolagus cuniculus OX=9986 GN=PGAM2 PE=1 SV=1
77	1	Custom	tr|A0A5F9DSZ2|A0A5F9DSZ2_RABIT	1000	0.16	38055	0.1	16	2	15-oxoprostaglandin 13-reductase OS=Oryctolagus cuniculus OX=9986 GN=PTGR1 PE=3 SV=1
78	1	Custom	tr|A0A5F9CP41|A0A5F9CP41_RABIT	975	5.58	8689	0.69	43	5	Ubiquitin-like domain-containing protein OS=Oryctolagus cuniculus OX=9986 PE=4 SV=1
79	1	Custom	tr|A0A5F9CCV6|A0A5F9CCV6_RABIT	937	0.15	81346	0.1	17	4	Post-proline cleaving enzyme OS=Oryctolagus cuniculus OX=9986 GN=PREP PE=3 SV=1
80	1	Custom	tr|A0A5F9DNF9|A0A5F9DNF9_RABIT	937	0.12	102381	0.09	17	4	Aminopeptidase OS=Oryctolagus cuniculus OX=9986 GN=NPEPPS PE=3 SV=1
81	1	Custom	tr|A0A5F9C3B5|A0A5F9C3B5_RABIT	934	0.06	48692	0.03	13	1	Heterogeneous nuclear ribonucleoprotein K OS=Oryctolagus cuniculus OX=9986 PE=4 SV=1
82	1	Custom	tr|A0A5S8H2S7|A0A5S8H2S7_RABIT	914	0.61	48448	0.25	20	8	Aspartate transaminase OS=Oryctolagus cuniculus OX=9986 GN=GOT2 PE=3 SV=1
83	1	Custom	tr|G1U8C4|G1U8C4_RABIT	872	0.22	28756	0.1	13	2	Proteasome activator subunit 1 OS=Oryctolagus cuniculus OX=9986 GN=PSME1 PE=3 SV=1
84	1	Custom	tr|G1TBS1|G1TBS1_RABIT	856	1.02	20098	0.26	23	5	Maillard deglycase OS=Oryctolagus cuniculus OX=9986 GN=PARK7 PE=3 SV=1
85	1	Custom	tr|G1TY57|G1TY57_RABIT	768	0.08	37037	0.03	22	1	Uncharacterized protein OS=Oryctolagus cuniculus OX=9986 PE=4 SV=2
86	1	Custom	tr|A0A5F9CW87|A0A5F9CW87_RABIT	766	1.52	12038	0.32	20	3	Thioredoxin OS=Oryctolagus cuniculus OX=9986 PE=3 SV=1
87	1	Custom	tr|A0A5F9CFP1|A0A5F9CFP1_RABIT	743	0.31	53675	0.19	26	5	Alpha-1B-glycoprotein OS=Oryctolagus cuniculus OX=9986 GN=A1BG PE=4 SV=1
88	1	Custom	tr|G1U1Q8|G1U1Q8_RABIT	739	0.25	12522	0.18	13	1	Macrophage migration inhibitory factor OS=Oryctolagus cuniculus OX=9986 GN=MIF PE=3 SV=1
89	1	Custom	tr|A0A5F9DV00|A0A5F9DV00_RABIT	736	0.25	25724	0.12	19	2	Platelet activating factor acetylhydrolase 1b catalytic subunit 2 OS=Oryctolagus cuniculus OX=9986 GN=PAFAH1B2 PE=4 SV=1
90	1	Custom	tr|G1SEK8|G1SEK8_RABIT	679	0.28	46464	0.1	19	4	Fetuin B OS=Oryctolagus cuniculus OX=9986 GN=FETUB PE=4 SV=2
91	1	Custom	tr|A0A5F9C975|A0A5F9C975_RABIT	676	0.4	16837	0.1	25	1	Ig-like domain-containing protein OS=Oryctolagus cuniculus OX=9986 PE=4 SV=1
92	1	Custom	tr|G1THL2|G1	610	0.75	20188	0.37	18	4	Ferritin OS=Oryctolagus cuniculus OX=9986 GN=FTL

			THL2_RABIT							PE=3 SV=2
93	1	Custom	tr|G1TME7|G1TME7_RABIT	575	0.83	9026	0.39	16	2	Cystatin B OS=Oryctolagus cuniculus OX=9986 GN=CSTB PE=3 SV=1
94	1	Custom	tr|A0A5F9CAW8|A0A5F9CAW8_RABIT	574	0.04	278518	0.03	11	4	Spectrin alpha, non-erythrocytic 1 OS=Oryctolagus cuniculus OX=9986 GN=SPTAN1 PE=4 SV=1
95	1	Custom	tr|A0A5F9D5A4|A0A5F9D5A4_RABIT	572	0.34	19235	0.16	13	2	RAB1A, member RAS oncogene family OS=Oryctolagus cuniculus OX=9986 GN=RAB1A PE=4 SV=1
96	1	Custom	tr|G1TKH3|G1TKH3_RABIT	545	1.04	15773	0.55	18	3	Superoxide dismutase [Cu-Zn] OS=Oryctolagus cuniculus OX=9986 GN=SOD1 PE=3 SV=1
97	1	Custom	tr|G1T2L1|G1T2L1_RABIT	523	0.25	25996	0.17	11	2	Proteasome subunit alpha type OS=Oryctolagus cuniculus OX=9986 PE=3 SV=1
98	1	Custom	tr|G1T918|G1T918_RABIT	463	0.35	29016	0.15	11	3	Proteasome subunit beta OS=Oryctolagus cuniculus OX=9986 GN=PSMB4 PE=3 SV=1
99	1	Custom	tr|G1SM91|G1SM91_RABIT	463	0.07	43820	0.04	5	1	Fumarylacetoacetase OS=Oryctolagus cuniculus OX=9986 GN=FAH PE=3 SV=2
100	1	Custom	tr|G1STJ4|G1STJ4_RABIT	463	0.15	82945	0.09	13	4	Complement C3 alpha chain OS=Oryctolagus cuniculus OX=9986 PE=4 SV=3
101	1	Custom	tr|A0A5F9C901|A0A5F9C901_RABIT	434	0.06	53876	0.02	15	1	Bleomycin hydrolase OS=Oryctolagus cuniculus OX=9986 GN=BLMH PE=3 SV=1
102	1	Custom	sp|P62943|FKB1A_RABIT	423	0.59	12000	0.29	13	2	Peptidyl-prolyl cis-trans isomerase FKBP1A OS=Oryctolagus cuniculus OX=9986 GN=FKBP1A PE=1 SV=2
103	1	Custom	tr|A0A5F9CLA2|A0A5F9CLA2_RABIT	416	0.19	16173	0.1	6	1	Ig-like domain-containing protein OS=Oryctolagus cuniculus OX=9986 PE=4 SV=1
104	1	Custom	tr|G1TCW5|G1TCW5_RABIT	412	0.12	50731	0.06	9	2	Peptidase D OS=Oryctolagus cuniculus OX=9986 GN=PEPD PE=4 SV=2
105	1	Custom	tr|A0A5F9DTZ9|A0A5F9DTZ9_RABIT	358	0.1	29362	0.05	5	1	Inositol polyphosphate-1-phosphatase OS=Oryctolagus cuniculus OX=9986 GN=INPP1 PE=3 SV=1
106	1	Custom	tr|G1SXW8|G1SXW8_RABIT	337	0.06	46180	0.02	7	1	Serpin family I member 2 OS=Oryctolagus cuniculus OX=9986 GN=SERPINI2 PE=3 SV=1
107	1	Custom	tr|G1SWI7|G1SWI7_RABIT	337	0.08	38869	0.03	6	1	Proteasome 20S subunit alpha 8 OS=Oryctolagus cuniculus OX=9986 GN=PSMA8 PE=3 SV=3
108	1	Custom	sp|P01683|KV02_RABIT	330	0.26	12233	0.1	7	1	Ig kappa chain V region 3315 OS=Oryctolagus cuniculus OX=9986 PE=1 SV=1
109	1	Custom	tr|A0A5F9C295|A0A5F9C295_RABIT	326	0.28	23078	0.12	5	2	Proteasome subunit alpha type OS=Oryctolagus cuniculus OX=9986 GN=PSMA6 PE=3 SV=1
110	1	Custom	tr|G1T4X8|G1T4X8_RABIT	326	0.86	22980	0.39	13	4	Proteasome subunit beta OS=Oryctolagus cuniculus OX=9986 GN=PSMB2 PE=3 SV=2

111	1	Custom	tr|G1T7Z6|G1T7Z6_RABIT	325	0.14	45003	0.08	13	2	Phosphoglycerate kinase OS=Oryctolagus cuniculus OX=9986 PE=3 SV=2
112	1	Custom	tr|G1SR03|G1SR03_RABIT	319	0.1	89950	0.06	7	3	15S Mg(2+)-ATPase p97 subunit OS=Oryctolagus cuniculus OX=9986 GN=VCP PE=3 SV=1
113	1	Custom	tr|G1T670|G1T670_RABIT	317	0.38	26549	0.19	11	3	Proteasome subunit alpha type OS=Oryctolagus cuniculus OX=9986 GN=PSMA5 PE=3 SV=2
114	1	Custom	tr|G1SH63|G1SH63_RABIT	309	0.06	52316	0.03	5	1	Glutathione synthetase OS=Oryctolagus cuniculus OX=9986 GN=GSS PE=3 SV=2
115	1	Custom	tr|A0A5F9CTP9|A0A5F9CTP9_RABIT	285	0.48	14363	0.18	4	2	Nucleoside-diphosphate kinase OS=Oryctolagus cuniculus OX=9986 PE=3 SV=1
116	1	Custom	tr|G1SN67|G1SN67_RABIT	283	0.31	21200	0.09	9	2	SERPIN domain-containing protein OS=Oryctolagus cuniculus OX=9986 PE=3 SV=2
117	1	Custom	tr|A0A5F9CRP0|A0A5F9CRP0_RABIT	282	0.37	26998	0.11	10	3	Chloride intracellular channel protein OS=Oryctolagus cuniculus OX=9986 PE=3 SV=1
118	1	Custom	tr|A0A5F9C4D8|A0A5F9C4D8_RABIT	281	0.22	57772	0.12	7	4	UTP--glucose-1-phosphate uridylyltransferase OS=Oryctolagus cuniculus OX=9986 PE=3 SV=1
119	1	Custom	tr|G1SZ14|G1SZ14_RABIT	281	0.08	36740	0.04	5	1	Proteasome subunit alpha type-3 OS=Oryctolagus cuniculus OX=9986 GN=PSMA3 PE=3 SV=2
120	1	Custom	tr|G1SH26|G1SH26_RABIT	279	0.08	71985	0.06	8	2	Uncharacterized protein OS=Oryctolagus cuniculus OX=9986 PE=4 SV=3
121	1	Custom	tr|G1T432|G1T432_RABIT	274	0.12	51871	0.09	4	2	Adenylyl cyclase-associated protein OS=Oryctolagus cuniculus OX=9986 PE=3 SV=1
122	1	Custom	tr|G1SHW8|G1SHW8_RABIT	273	0.11	57092	0.07	9	2	Keratin 85 OS=Oryctolagus cuniculus OX=9986 GN=KRT85 PE=3 SV=2
123	1	Custom	tr|A0A5F9DPL1|A0A5F9DPL1_RABIT	270	0.17	36840	0.06	6	2	L-lactate dehydrogenase OS=Oryctolagus cuniculus OX=9986 GN=LDHB PE=3 SV=1
124	1	Custom	tr|A0A5F9C494|A0A5F9C494_RABIT	270	0.07	43100	0.03	8	1	Malate dehydrogenase, mitochondrial OS=Oryctolagus cuniculus OX=9986 GN=MDH2 PE=3 SV=1
125	1	Custom	tr|A0A5F9DJ89|A0A5F9DJ89_RABIT	268	0.47	14498	0.21	5	2	2-iminobutanoate/2-iminopropanoate deaminase OS=Oryctolagus cuniculus OX=9986 PE=3 SV=1
126	1	Custom	tr|A0A5F9C3R6|A0A5F9C3R6_RABIT	257	0.19	33421	0.1	10	2	Asparaginase-like protein 1 OS=Oryctolagus cuniculus OX=9986 GN=ASRGL1 PE=3 SV=1
127	1	Custom	tr|A0A5F9CYX6|A0A5F9CYX6_RABIT	251	0.19	15880	0.06	10	1	Ig-like domain-containing protein OS=Oryctolagus cuniculus OX=9986 PE=4 SV=1
128	1	Custom	tr|G1SJ23|G1SJ23_RABIT	249	0.04	75020	0.03	6	1	Calcium-activated neutral proteinase 1 OS=Oryctolagus cuniculus OX=9986 GN=CAPN1 PE=3 SV=2

129	1	Custom	tr|A0A5F9CPK4|A0A5F9CPK4_RABIT	242	0.07	41022	0.04	4	1	Proteasome endopeptidase complex OS=Oryctolagus cuniculus OX=9986 GN=PSMB1 PE=4 SV=1
130	1	Custom	tr|A0A5F9C239|A0A5F9C239_RABIT	226	0.08	39168	0.03	12	1	Uncharacterized protein OS=Oryctolagus cuniculus OX=9986 PE=4 SV=1
131	1	Custom	tr|A0A5F9C1C3|A0A5F9C1C3_RABIT	224	0.09	33488	0.05	3	1	Thioredoxin domain-containing protein OS=Oryctolagus cuniculus OX=9986 PE=4 SV=1
132	1	Custom	tr|A0A5F9D4Q9|A0A5F9D4Q9_RABIT	222	0.17	18021	0.14	4	1	Ubiquitin conjugating enzyme E2 L5 OS=Oryctolagus cuniculus OX=9986 PE=3 SV=1
133	1	Custom	tr|A0A5F9CHA6|A0A5F9CHA6_RABIT	219	0.1	29417	0.07	11	1	Ig-like domain-containing protein OS=Oryctolagus cuniculus OX=9986 PE=4 SV=1
134	1	Custom	tr|A0A5F9C1C5|A0A5F9C1C5_RABIT	217	0.04	161930	0.02	4	2	Uncharacterized protein OS=Oryctolagus cuniculus OX=9986 PE=3 SV=1
135	1	Custom	tr|G1THE8|G1THE8_RABIT	211	0.23	13186	0.09	6	1	Ig-like domain-containing protein OS=Oryctolagus cuniculus OX=9986 PE=4 SV=2
136	1	Custom	tr|G1TIY2|G1TIY2_RABIT	210	0.18	16645	0.07	5	1	Ig-like domain-containing protein OS=Oryctolagus cuniculus OX=9986 PE=4 SV=3
137	1	Custom	tr|G1TM35|G1TM35_RABIT	208	0.13	22894	0.05	3	1	Fission, mitochondrial 1 OS=Oryctolagus cuniculus OX=9986 GN=FIS1 PE=3 SV=3
138	1	Custom	tr|A0A5F9D4A7|A0A5F9D4A7_RABIT	202	0.06	53676	0.04	4	1	SERPIN domain-containing protein OS=Oryctolagus cuniculus OX=9986 GN=SERPINF2 PE=3 SV=1
139	1	Custom	sp|Q8WN94|ACBP_RABIT	199	0.32	9909	0.11	8	1	Acyl-CoA-binding protein OS=Oryctolagus cuniculus OX=9986 GN=DBI PE=3 SV=3
140	1	Custom	tr|G1SI83|G1SI83_RABIT	198	1.09	11195	0.29	7	3	S100 calcium binding protein A13 OS=Oryctolagus cuniculus OX=9986 GN=S100A13 PE=4 SV=1
141	1	Custom	tr|G1TI72|G1TI72_RABIT	194	0.12	50108	0.02	10	1	TBC1 domain family member 10C OS=Oryctolagus cuniculus OX=9986 GN=TBC1D10C PE=4 SV=3
142	1	Custom	tr|A0A5F9C531|A0A5F9C531_RABIT	188	0.16	39621	0.07	11	2	Glycogenin-1 OS=Oryctolagus cuniculus OX=9986 GN=GYG1 PE=4 SV=1
143	1	Custom	tr|A0A5F9C1I9|A0A5F9C1I9_RABIT	180	0.14	44156	0.05	21	2	Actin-like protein 3 OS=Oryctolagus cuniculus OX=9986 PE=3 SV=1
144	1	Custom	tr|A0A5F9D3N8|A0A5F9D3N8_RABIT	179	0.08	107260	0.06	8	3	Gal_mutarotas_2 domain-containing protein OS=Oryctolagus cuniculus OX=9986 PE=3 SV=1
145	1	Custom	tr|A0A5S8HWD2|A0A5S8H	176	0.28	11231	0.08	6	1	Protein S100-A6 OS=Oryctolagus cuniculus OX=9986 PE=4 SV=1

			WD2_RABIT							
146	1	Custom	tr|A0A5F9DLC0|A0A5F9DLC0_RABIT	167	0.06	48027	0.04	2	1	Creatine kinase OS=Oryctolagus cuniculus OX=9986 PE=3 SV=1
147	1	Custom	tr|A0A5F9DUX5|A0A5F9DUX5_RABIT	166	0.34	9288	0.12	3	1	SH3 domain binding glutamate rich protein like 3 OS=Oryctolagus cuniculus OX=9986 GN=SH3BGRL3 PE=3 SV=1
148	1	Custom	tr|A0A5S8H3Z9|A0A5S8H3Z9_RABIT	163	0.17	36269	0.07	3	2	Alpha-1-acid glycoprotein OS=Oryctolagus cuniculus OX=9986 PE=3 SV=1
149	1	Custom	tr|G1SN70|G1SN70_RABIT	163	0.31	43188	0.2	6	4	Serpin family B member 9 OS=Oryctolagus cuniculus OX=9986 GN=SERPINB9 PE=3 SV=3
150	1	Custom	tr|A0A5F9DEL1|A0A5F9DEL1_RABIT	147	0.47	14665	0.22	5	2	Ig-like domain-containing protein OS=Oryctolagus cuniculus OX=9986 PE=4 SV=1
151	1	Custom	tr|G1SQR7|G1SQR7_RABIT	137	0.77	14640	0.33	9	3	NTF2 domain-containing protein OS=Oryctolagus cuniculus OX=9986 PE=4 SV=1
152	1	Custom	tr|G1TA78|G1TA78_RABIT	135	0.05	55377	0.03	8	1	T1-TrpRS OS=Oryctolagus cuniculus OX=9986 GN=WARS1 PE=3 SV=2
153	1	Custom	tr|A0A5F9CM20|A0A5F9CM20_RABIT	135	0.05	264213	0.03	4	4	Talin 1 OS=Oryctolagus cuniculus OX=9986 GN=TLN1 PE=4 SV=1
154	1	contaminants	Q3T052	127	0.03	101620	0.01	12	1	TREMBL:Q3T052;Q5EA67 (Bos taurus) Inter-alpha (Globulin) inhibitor H4
154	2	Custom	tr|A0A5F9DAJ6|A0A5F9DAJ6_RABIT	94	0.14	21122	0.04	11	1	Mediator of RNA polymerase II transcription subunit 6 OS=Oryctolagus cuniculus OX=9986 GN=MED6 PE=3 SV=1
155	1	Custom	tr|G1TM88|G1TM88_RABIT	122	0.06	46013	0.02	3	1	Serpin family A member 3 OS=Oryctolagus cuniculus OX=9986 GN=SERPINA3 PE=3 SV=2
156	1	Custom	tr|G1T519|G1T519_RABIT	120	0.1	30377	0.03	5	1	Proteasome subunit alpha type OS=Oryctolagus cuniculus OX=9986 GN=PSMA4 PE=3 SV=3
157	1	Custom	sp|P41982|SODM_RABIT	114	0.13	22755	0.07	4	1	Superoxide dismutase [Mn], mitochondrial (Fragment) OS=Oryctolagus cuniculus OX=9986 GN=SOD2 PE=2 SV=1
158	1	Custom	tr|G1TI63|G1TI63_RABIT	109	0.23	13399	0.06	6	1	Ig-like domain-containing protein OS=Oryctolagus cuniculus OX=9986 PE=4 SV=2
159	1	Custom	tr|A0A5F9CI66|A0A5F9CI66_RABIT	106	0.02	125209	0.01	2	1	Ceruloplasmin OS=Oryctolagus cuniculus OX=9986 GN=CP PE=3 SV=1
160	1	contaminants	Q2UVX4	103	0.02	188789	0.01	2	1	SWISS-PROT:Q2UVX4 (Bos taurus) Complement C3 precursor
161	1	Custom	tr|G1SIK0|G1SIK0_RABIT	100	0.18	53059	0.08	5	3	Antithrombin-III OS=Oryctolagus cuniculus OX=9986 GN=SERPINC1 PE=3 SV=1
162	1	Custom	tr|A0A5F9CUC6|A0A5F9CUC	98	0.18	34426	0.12	5	2	Arp2/3 complex 34 kDa subunit OS=Oryctolagus cuniculus OX=9986 GN=ARPC2 PE=3 SV=1

			6_RABIT							
163	1	Custom	tr|G1SQ46|G1SQ46_RABIT	96	0.28	22865	0.23	4	2	Rho GDP dissociation inhibitor beta OS=Oryctolagus cuniculus OX=9986 GN=ARHGDIB PE=3 SV=1
164	1	Custom	tr|A0A5F9CHU5|A0A5F9CHU5_RABIT	95	0.25	25360	0.19	2	2	Uncharacterized protein OS=Oryctolagus cuniculus OX=9986 PE=4 SV=1
165	1	Custom	tr|A0A5F9CD55|A0A5F9CD55_RABIT	94	0.11	57636	0.1	2	2	Melanoma cell adhesion molecule OS=Oryctolagus cuniculus OX=9986 GN=MCAM PE=4 SV=1
166	1	Custom	tr|A0A5F9DNV0|A0A5F9DNV0_RABIT	93	0.03	174711	0.03	4	2	C4a anaphylatoxin OS=Oryctolagus cuniculus OX=9986 PE=4 SV=1
167	1	Custom	tr|A0A5F9C5Y6|A0A5F9C5Y6_RABIT	88	0.08	35632	0.04	1	1	Parvin alpha OS=Oryctolagus cuniculus OX=9986 GN=PARVA PE=3 SV=1
168	1	Custom	tr|A0A5F9CTS6|A0A5F9CTS6_RABIT	86	0.04	79476	0.04	2	1	Uncharacterized protein OS=Oryctolagus cuniculus OX=9986 PE=4 SV=1
169	1	Custom	tr|A0A5F9DI38|A0A5F9DI38_RABIT	84	0.1	29222	0.03	3	1	Heterogeneous nuclear ribonucleoprotein A/B OS=Oryctolagus cuniculus OX=9986 GN=HNRNPAB PE=4 SV=1
170	1	Custom	tr|G1U8T9|G1U8T9_RABIT	84	0.07	41473	0.03	2	1	Potassium channel tetramerization domain containing 12 OS=Oryctolagus cuniculus OX=9986 GN=KCTD12 PE=4 SV=2
171	1	Custom	tr|G1TAF5|G1TAF5_RABIT	80	0.12	25892	0.04	2	1	Mammalian ependymin-related protein 1 OS=Oryctolagus cuniculus OX=9986 GN=EPDR1 PE=3 SV=2
172	1	Custom	tr|A0A5F9CXV9|A0A5F9CXV9_RABIT	79	0.39	8163	0.17	1	1	Rad60-SLD domain-containing protein OS=Oryctolagus cuniculus OX=9986 PE=4 SV=1
173	1	Custom	tr|A0A5F9DBK7|A0A5F9DBK7_RABIT	79	0.13	24188	0.05	2	1	Hypoxanthine phosphoribosyltransferase OS=Oryctolagus cuniculus OX=9986 GN=HPRT1 PE=3 SV=1
174	1	Custom	tr|G1SRF7|G1SRF7_RABIT	77	0.04	73822	0.02	1	1	75 kDa glucose-regulated protein OS=Oryctolagus cuniculus OX=9986 GN=HSPA9 PE=3 SV=1
175	1	Custom	tr|G1SQG6|G1SQG6_RABIT	76	0.05	63588	0.03	1	1	SERPIN domain-containing protein OS=Oryctolagus cuniculus OX=9986 PE=3 SV=3
176	1	Custom	tr|G1SJI2|G1SJI2_RABIT	76	0.07	45306	0.04	1	1	Indoleamine 2,3-dioxygenase 1 OS=Oryctolagus cuniculus OX=9986 GN=IDO1 PE=3 SV=3
177	1	Custom	tr|A0A5F9CAE5|A0A5F9CAE5_RABIT	76	0.04	67910	0.04	3	1	Thioredoxin-disulfide reductase OS=Oryctolagus cuniculus OX=9986 GN=TXNRD1 PE=3 SV=1
178	1	Custom	tr|G1TYT3|G1TYT3_RABIT	76	0.03	98489	0.03	1	1	Cadherin-1 OS=Oryctolagus cuniculus OX=9986 GN=CDH1 PE=4 SV=3
179	1	Custom	sp|Q8MJF1|MI	75	0.09	34239	0.05	1	1	Mimecan OS=Oryctolagus cuniculus OX=9986 GN=OGN

			ME_RABIT							PE=1 SV=1
180	1	Custom	tr|G1STT3|G1STT3_RABIT	74	0.13	45654	0.08	3	2	Serpin family B member 10 OS=Oryctolagus cuniculus OX=9986 GN=SERPINB10 PE=3 SV=1
181	1	Custom	tr|A0A5F9CXT8|A0A5F9CXT8_RABIT	72	0.13	47137	0.05	4	2	Isocitrate dehydrogenase [NADP] OS=Oryctolagus cuniculus OX=9986 GN=IDH1 PE=3 SV=1
182	1	Custom	tr|A0A5F9C5W1|A0A5F9C5W1_RABIT	68	0.14	22299	0.03	4	1	Meiotic nuclear division protein 1 homolog OS=Oryctolagus cuniculus OX=9986 PE=3 SV=1
183	1	Custom	tr|A0A5F9D5Z5|A0A5F9D5Z5_RABIT	66	0.06	48819	0.03	1	1	Alpha-galactosidase OS=Oryctolagus cuniculus OX=9986 GN=NAGA PE=3 SV=1
184	1	Custom	tr|G1SR28|G1SR28_RABIT	65	0.12	25905	0.04	2	1	Platelet activating factor acetylhydrolase 1b catalytic subunit 3 OS=Oryctolagus cuniculus OX=9986 GN=PAFAH1B3 PE=4 SV=1
185	1	Custom	tr|G1TD98|G1TD98_RABIT	64	0.06	51137	0.06	3	1	Glutathione-disulfide reductase OS=Oryctolagus cuniculus OX=9986 GN=GSR PE=3 SV=2
186	1	Custom	tr|G1T090|G1T090_RABIT	64	0.04	83081	0.04	2	1	Dipeptidyl peptidase 3 OS=Oryctolagus cuniculus OX=9986 GN=DPP3 PE=3 SV=2
187	1	Custom	tr|A0A5F9DCJ0|A0A5F9DCJ0_RABIT	62	0.2	31717	0.14	3	2	Crystallin zeta OS=Oryctolagus cuniculus OX=9986 GN=CRYZ PE=3 SV=1
188	1	Custom	tr|A0A5F9DGR9|A0A5F9DGR9_RABIT	61	0.03	84577	0.02	6	1	SRCR domain-containing protein OS=Oryctolagus cuniculus OX=9986 PE=3 SV=1
189	1	Custom	tr|A0A5F9D9F5|A0A5F9D9F5_RABIT	61	0.05	58539	0.01	6	1	RCC1 and BTB domain containing protein 1 OS=Oryctolagus cuniculus OX=9986 GN=RCBTB1 PE=4 SV=1
190	1	Custom	tr|G1SHV9|G1SHV9_RABIT	61	0.13	23249	0.09	1	1	Proteasome subunit beta OS=Oryctolagus cuniculus OX=9986 GN=PSMB3 PE=3 SV=2
191	1	Custom	tr|G1SK20|G1SK20_RABIT	58	0.05	54118	0.02	2	1	Clathrin heavy chain linker domain-containing protein 1 OS=Oryctolagus cuniculus OX=9986 PE=4 SV=2
192	1	Custom	tr|A0A5F9CXZ6|A0A5F9CXZ6_RABIT	56	0.2	31526	0.16	2	2	Ras suppressor protein 1 OS=Oryctolagus cuniculus OX=9986 GN=RSU1 PE=4 SV=1
193	1	Custom	tr|G1SQF0|G1SQF0_RABIT	54	0.13	47958	0.07	4	2	Uncharacterized protein OS=Oryctolagus cuniculus OX=9986 PE=3 SV=2
194	1	Custom	tr|A0A5F9C3S2|A0A5F9C3S2_RABIT	50	0.11	28015	0.09	4	1	Esterase D OS=Oryctolagus cuniculus OX=9986 PE=3 SV=1
195	1	Custom	tr|A0A5F9C8M0|A0A5F9C8M0_RABIT	50	0.03	235323	0.01	11	2	HEAT repeat-containing protein 1 OS=Oryctolagus cuniculus OX=9986 GN=HEATR1 PE=3 SV=1
196	1	Custom	tr|G1T0Z8|G1	48	0.18	17344	0.12	1	1	Prefoldin subunit 5 OS=Oryctolagus cuniculus OX=9986

			T0Z8_RABIT							GN=PFDN5 PE=3 SV=2
197	1	Custom	tr|A0A5F9DE89|A0A5F9DE89_RABIT	45	0.04	70377	0.04	1	1	Afamin OS=Oryctolagus cuniculus OX=9986 GN=AFM PE=4 SV=1
198	1	Custom	tr|G1TDT6|G1TDT6_RABIT	44	0.08	39537	0.06	2	1	Isochorismatase domain containing 1 OS=Oryctolagus cuniculus OX=9986 GN=ISOC1 PE=3 SV=3
199	1	Custom	tr|A0A5F9CGS0|A0A5F9CGS0_RABIT	42	0.07	42620	0.03	4	1	STE20 related adaptor beta OS=Oryctolagus cuniculus OX=9986 GN=STRADB PE=4 SV=1
200	1	Custom	tr|A0A5F9C3J7|A0A5F9C3J7_RABIT	42	0.21	14379	0.05	5	1	Uncharacterized protein OS=Oryctolagus cuniculus OX=9986 PE=4 SV=1
201	1	Custom	tr|G1TLT6|G1TLT6_RABIT	42	0.03	109633	0.01	5	1	Ankyrin repeat domain 35 OS=Oryctolagus cuniculus OX=9986 GN=ANKRD35 PE=4 SV=2
202	1	Custom	tr|A0A5F9CNT6|A0A5F9CNT6_RABIT	41	0.07	42383	0.02	5	1	Septin 12 OS=Oryctolagus cuniculus OX=9986 GN=SEPTIN12 PE=3 SV=1
203	1	Custom	tr|G1SMW6|G1SMW6_RABIT	41	0.05	59498	0.02	6	1	Occludin OS=Oryctolagus cuniculus OX=9986 GN=OCLN PE=3 SV=1
204	1	Custom	tr|A0A5F9CKR5|A0A5F9CKR5_RABIT	40	0.09	34626	0.1	1	1	Serine/threonine-protein phosphatase 2A activator OS=Oryctolagus cuniculus OX=9986 PE=3 SV=1
205	1	Custom	tr|A0A5F9C3R8|A0A5F9C3R8_RABIT	40	0.16	18938	0.07	1	1	RAP1B, member of RAS oncogene family OS=Oryctolagus cuniculus OX=9986 PE=4 SV=1
206	1	Custom	tr|A0A5F9D3K0|A0A5F9D3K0_RABIT	38	0.03	103485	0.01	2	1	Cullin 4B OS=Oryctolagus cuniculus OX=9986 GN=CUL4B PE=3 SV=1
207	1	contaminants	ENSEMBL:ENSBTAP00000032840	36	0.03	92957	0.01	2	1	(Bos taurus) similar to apolipoprotein B, partial
208	1	Custom	tr|A0A5F9DQN1|A0A5F9DQN1_RABIT	36	0.06	47989	0.01	10	1	PRP18 homolog OS=Oryctolagus cuniculus OX=9986 PE=3 SV=1
209	1	Custom	tr|A0A5F9CH47|A0A5F9CH47_RABIT	35	0.04	81577	0.01	7	1	Catenin beta 1 OS=Oryctolagus cuniculus OX=9986 GN=CTNNB1 PE=3 SV=1
210	1	Custom	tr|G1SEK2|G1SEK2_RABIT	35	0.06	48967	0.06	1	1	Protein phosphatase, Mg2+/Mn2+ dependent 1F OS=Oryctolagus cuniculus OX=9986 GN=PPM1F PE=3 SV=3
211	1	Custom	tr|A0A5F9DLL2|A0A5F9DLL2_RABIT	35	0.1	28869	0.05	4	1	t-SNARE coiled-coil homology domain-containing protein OS=Oryctolagus cuniculus OX=9986 PE=4 SV=1
212	1	Custom	tr|G1TDX5|G1	34	0.05	64572	0.01	1	1	Uncharacterized protein OS=Oryctolagus cuniculus

			TDX5_RABIT							OX=9986 PE=4 SV=3
213	1	Custom	tr|G1SQU1|G1SQU1_RABIT	34	0.1	29399	0.07	3	1	Proteasome subunit beta OS=Oryctolagus cuniculus OX=9986 GN=PSMB10 PE=3 SV=3
214	1	Custom	tr|A0A5F9CH62|A0A5F9CH62_RABIT	34	0.08	36193	0.03	1	1	Uncharacterized protein OS=Oryctolagus cuniculus OX=9986 PE=3 SV=1
215	1	Custom	tr|A0A5F9CHF0|A0A5F9CHF0_RABIT	34	0.04	71209	0.02	1	1	Kininogen 1 OS=Oryctolagus cuniculus OX=9986 GN=KNG1 PE=4 SV=1
216	1	Custom	tr|A0A5F9CTF6|A0A5F9CTF6_RABIT	33	0.01	280843	0.01	1	1	Centrosomal protein 295 OS=Oryctolagus cuniculus OX=9986 GN=CEP295 PE=4 SV=1
217	1	Custom	tr|G1TYE2|G1TYE2_RABIT	32	0.12	25804	0.08	2	1	Latexin OS=Oryctolagus cuniculus OX=9986 GN=LXN PE=3 SV=1
218	1	Custom	tr|A0A5F9CG66|A0A5F9CG66_RABIT	32	0.04	66092	0.01	2	1	E74 like ETS transcription factor 1 OS=Oryctolagus cuniculus OX=9986 GN=ELF1 PE=3 SV=1
219	1	Custom	tr|G1TUP6|G1TUP6_RABIT	32	0.08	36645	0.02	1	1	Leucine rich repeat containing 75A OS=Oryctolagus cuniculus OX=9986 GN=LRRC75A PE=4 SV=2
220	1	Custom	tr|A0A5F9CMD9|A0A5F9CMD9_RABIT	31	0.03	101625	0.01	1	1	Zinc finger protein 574 OS=Oryctolagus cuniculus OX=9986 GN=ZNF574 PE=4 SV=1
221	1	Custom	tr|G1TMZ2|G1TMZ2_RABIT	31	0.1	29811	0.02	7	1	Ribosomal_S7 domain-containing protein OS=Oryctolagus cuniculus OX=9986 PE=3 SV=2
222	1	Custom	tr|A0A5F9CAI2|A0A5F9CAI2_RABIT	30	0.02	162482	0.01	2	1	Adhesion G protein-coupled receptor L3 OS=Oryctolagus cuniculus OX=9986 GN=ADGRL3 PE=4 SV=1
223	1	Custom	tr|G1SPA2|G1SPA2_RABIT	30	0.01	256836	0	1	1	Zonadhesin OS=Oryctolagus cuniculus OX=9986 GN=ZAN PE=4 SV=2
224	1	Custom	tr|A0A5F9CEL3|A0A5F9CEL3_RABIT	29	0.05	64392	0.02	1	1	Integrase OS=Oryctolagus cuniculus OX=9986 PE=4 SV=1
225	1	Custom	tr|A0A5F9CDJ4|A0A5F9CDJ4_RABIT	28	0.06	46894	0.02	1	1	Acyl-protein thioesterase 1 OS=Oryctolagus cuniculus OX=9986 GN=LYPLA1 PE=3 SV=1
226	1	Custom	tr|A0A5F9C2W4|A0A5F9C2W4_RABIT	28	0.2	15509	0.04	2	1	Cathepsin C OS=Oryctolagus cuniculus OX=9986 GN=CTSC PE=4 SV=1
227	1	Custom	tr|G1TNT9|G1TNT9_RABIT	28	0.03	95115	0.01	1	1	CLPTM1 regulator of GABA type A receptor forward trafficking OS=Oryctolagus cuniculus OX=9986 GN=CLPTM1 PE=3 SV=3
228	1	Custom	tr|G1SKP6|G1SKP6_RABIT	28	0.04	76702	0.01	2	1	Leucine rich repeat, Ig-like and transmembrane domains 3 OS=Oryctolagus cuniculus OX=9986 GN=LRIT3 PE=4 SV=3

229	1	Custom	tr|A0A5F9DCZ7|A0A5F9DCZ7_RABIT	28	0.05	54410	0.02	2	1	Chromosome 12 open reading frame 56 OS=Oryctolagus cuniculus OX=9986 GN=C12orf56 PE=4 SV=1
230	1	Custom	tr|A0A5F9CIV6|A0A5F9CIV6_RABIT	28	0.02	129214	0	1	1	Potassium sodium-activated channel subfamily T member 2 OS=Oryctolagus cuniculus OX=9986 GN=KCNT2 PE=4 SV=1
231	1	Custom	tr|G1T616|G1T616_RABIT	27	0.04	84002	0.02	1	1	Phosphoglucomutase 2 OS=Oryctolagus cuniculus OX=9986 GN=PGM2 PE=3 SV=3
232	1	Custom	tr|G1T4M3|G1T4M3_RABIT	27	0.05	60430	0.03	2	1	Arm_2 domain-containing protein OS=Oryctolagus cuniculus OX=9986 PE=3 SV=1
233	1	Custom	tr|A0A5F9CJP6|A0A5F9CJP6_RABIT	26	0.18	17453	0.14	1	1	Component 3 of promoter of RISC OS=Oryctolagus cuniculus OX=9986 GN=TSN PE=4 SV=1
234	1	Custom	tr|A0A5F9CQP0|A0A5F9CQP0_RABIT	26	0.22	13958	0.06	3	1	GLOBIN domain-containing protein OS=Oryctolagus cuniculus OX=9986 PE=3 SV=1
235	1	Custom	tr|A0A5F9CR62|A0A5F9CR62_RABIT	25	0.04	75978	0.05	1	1	Uncharacterized protein OS=Oryctolagus cuniculus OX=9986 PE=4 SV=1
236	1	Custom	tr|G1T2D1|G1T2D1_RABIT	25	0.02	135892	0.01	1	1	Mitogen-activated protein kinase binding protein 1 OS=Oryctolagus cuniculus OX=9986 GN=MAPKBP1 PE=4 SV=2
237	1	Custom	tr|G1SWW9|G1SWW9_RABIT	25	0.09	32680	0.04	2	1	Rho family GTPase 2 OS=Oryctolagus cuniculus OX=9986 GN=RND2 PE=4 SV=3
238	1	Custom	tr|A0A5F9CQW8|A0A5F9CQW8_RABIT	25	0.22	13760	0.07	1	1	Epididymal secretory protein E1 OS=Oryctolagus cuniculus OX=9986 GN=NPC2 PE=3 SV=1
239	1	Custom	tr|A0A5F9CAH1|A0A5F9CAH1_RABIT	25	0.02	161611	0.01	2	1	ATP binding cassette subfamily C member 5 OS=Oryctolagus cuniculus OX=9986 GN=ABCC5 PE=4 SV=1
240	1	Custom	tr|A0A5F9CTS5|A0A5F9CTS5_RABIT	25	0.11	28015	0.03	1	1	Uncharacterized protein OS=Oryctolagus cuniculus OX=9986 PE=3 SV=1
241	1	Custom	tr|G1SM24|G1SM24_RABIT	25	0	636692	0	1	1	Midasin OS=Oryctolagus cuniculus OX=9986 GN=MDN1 PE=3 SV=3
242	1	Custom	tr|A0A5F9CH54|A0A5F9CH54_RABIT	24	0.05	60928	0.02	1	1	N-acetylglucosamine-6-sulfatase OS=Oryctolagus cuniculus OX=9986 GN=GNS PE=3 SV=1
243	1	Custom	tr|A0A5F9D316|A0A5F9D316_RABIT	24	0.05	62385	0.05	1	1	Malic enzyme OS=Oryctolagus cuniculus OX=9986 GN=ME1 PE=3 SV=1
244	1	Custom	tr|A0A5F9CZD5|A0A5F9CZD5_RABIT	24	0.06	53156	0.02	1	1	Nuclear receptor subfamily 1 group C member 1 OS=Oryctolagus cuniculus OX=9986 GN=PPARA PE=3 SV=1

245	1	Custom	tr|A0A5F9C413|A0A5F9C413_RABIT	24	0.09	34916	0.03	1	1	GULP PTB domain containing engulfment adaptor 1 OS=Oryctolagus cuniculus OX=9986 GN=GULP1 PE=4 SV=1
246	1	Custom	tr|A0A5F9CE67|A0A5F9CE67_RABIT	24	0.03	104314	0.01	1	1	Uncharacterized protein OS=Oryctolagus cuniculus OX=9986 PE=4 SV=1
247	1	Custom	tr|A0A5F9DBZ8|A0A5F9DBZ8_RABIT	24	0.07	40286	0.05	1	1	Terminal uridylyl transferase 4 OS=Oryctolagus cuniculus OX=9986 GN=TUT4 PE=4 SV=1
248	1	Custom	tr|A0A5F9D2E7|A0A5F9D2E7_RABIT	24	0.03	94456	0.01	1	1	ADAM metallopeptidase domain 23 OS=Oryctolagus cuniculus OX=9986 GN=ADAM23 PE=4 SV=1
249	1	Custom	tr|G1TAH7|G1TAH7_RABIT	24	0.04	68341	0.01	1	1	Transketolase OS=Oryctolagus cuniculus OX=9986 GN=TKT PE=4 SV=2
250	1	Custom	tr|A0A5F9D5M7|A0A5F9D5M7_RABIT	23	0.06	53901	0.02	3	1	2-phosphoxylose phosphatase 1 OS=Oryctolagus cuniculus OX=9986 GN=PXYLP1 PE=4 SV=1
251	1	Custom	tr|G1SJH7|G1SJH7_RABIT	23	0.22	13727	0.13	1	1	Chromosome 11 open reading frame 71 OS=Oryctolagus cuniculus OX=9986 GN=C11orf71 PE=4 SV=1
252	1	Custom	tr|G1SNQ8|G1SNQ8_RABIT	22	0.05	61462	0.05	1	1	Beta-hexosaminidase OS=Oryctolagus cuniculus OX=9986 GN=HEXB PE=3 SV=3
253	1	Custom	tr|A0A5F9C427|A0A5F9C427_RABIT	22	0.03	89257	0.01	1	1	Uncharacterized protein OS=Oryctolagus cuniculus OX=9986 PE=4 SV=1
254	1	Custom	tr|G1T2W2|G1T2W2_RABIT	22	0.03	98091	0.02	1	1	DLG associated protein 3 OS=Oryctolagus cuniculus OX=9986 GN=DLGAP3 PE=3 SV=3
255	1	Custom	tr|G1SVF3|G1SVF3_RABIT	21	0.01	308337	0	2	1	Ubiquitin protein ligase E3 component n-recognin 5 OS=Oryctolagus cuniculus OX=9986 GN=UBR5 PE=4 SV=3
256	1	Custom	tr|A0A5F9DFZ8|A0A5F9DFZ8_RABIT	21	0.01	331654	0.01	1	1	ASH1 like histone lysine methyltransferase OS=Oryctolagus cuniculus OX=9986 GN=ASH1L PE=4 SV=1
257	1	Custom	tr|G1T1P8|G1T1P8_RABIT	21	0.06	47644	0.02	1	1	Methyltransferase-like protein OS=Oryctolagus cuniculus OX=9986 GN=METTL8 PE=3 SV=3
258	1	Custom	tr|A0A5F9DRG0|A0A5F9DRG0_RABIT	21	0.06	52855	0.02	1	1	Dynactin subunit 4 OS=Oryctolagus cuniculus OX=9986 GN=DCTN4 PE=4 SV=1
259	1	Custom	tr|A0A5F9CME5|A0A5F9CME5_RABIT	21	0.01	206362	0.01	1	1	Dedicator of cytokinesis 5 OS=Oryctolagus cuniculus OX=9986 GN=DOCK5 PE=3 SV=1
260	1	Custom	tr|G1U6P5|G1U6P5_RABIT	21	0.08	38905	0.07	1	1	Uncharacterized protein OS=Oryctolagus cuniculus OX=9986 PE=4 SV=2
261	1	Custom	tr|G1SZF5|G1SZF5_RABIT	21	0.05	59625	0.02	1	1	Natriuretic peptide receptor 3 OS=Oryctolagus cuniculus OX=9986 GN=NPR3 PE=4 SV=1
262	1	Custom	tr|A0A5F9C2D	21	0.16	18495	0.05	1	1	Uncharacterized protein OS=Oryctolagus cuniculus

			7|A0A5F9C2D7_RABIT							OX=9986 PE=4 SV=1
263	1	Custom	tr|A0A5F9CB39|A0A5F9CB39_RABIT	20	0.01	257049	0	1	1	Neuron navigator 2 OS=Oryctolagus cuniculus OX=9986 GN=NAV2 PE=3 SV=1
264	1	Custom	tr|G1TC80|G1TC80_RABIT	20	0.08	36148	0.03	1	1	Mesenteric estrogen dependent adipogenesis OS=Oryctolagus cuniculus OX=9986 GN=MEDAG PE=4 SV=2

Database 1: contaminants 20160129 (249 sequences; 128730 residues); database 2: Custom (41459 sequences; 24801325 residues); type of search: MS/MS Ion Search; enzyme: trypsin/P; fixed modifications: Carbamidomethyl (C); variable modifications: deamidated (NQ), oxidation (M); mass values: monoisotopic; protein mass:unrestricted; peptide mass tolerance: 3 ppm; fragment mass tolerance: 0.6 Da; max missed cleavages: 2; instrument type: ESI-TRAP; number of queries: 30645; significance threshold: $p < 0.05$; max. number of families: AUTO; ions score or expect cut-off: 20; preferred taxonomy: all entries.
