# Supplementary material for: Modulation of Cellular Senescence in HEK293 and HepG2 Cells by Ultrafiltrates UPla and ULu Is Partly Mediated by Modulation of Mitochondrial Homeostasis under Oxidative Stress
Source: Int J Mol Sci. 2023 Apr 4;24(7):6748. doi: 10.3390/ijms24076748 (PMC10095350; doi:10.3390/ijms24076748)
Supplement: Supplementary file 1 [file ijms-24-06748-s001.zip › Tables S3 and S4.pdf]

Table S3. Table with top 10 GO terms for all three GO trees: biological product (BP), cellular component (CC), and molecular function (MF) for UPla.

GO Tree		Term	Annotated	Significant	Expected	p-value	proteins
GO:0006096	BP	glycolytic process	49	4	0.27	0.00016	G1SPF5, G1T652, G1T7Z6, G1U7S4
GO:0034372	BP	very-low-density lipoprotein particle re...	5	2	0.03	0.00030	G1SZV5, B7NZM0
GO:0043691	BP	reverse cholesterol transport	5	2	0.03	0.00030	G1SZV5, B7NZM0
GO:0034435	BP	cholesterol esterification	6	2	0.03	0.00045	G1SZV5, B7NZM0
GO:0030300	BP	regulation of intestinal cholesterol abs...	6	2	0.03	0.00045	G1SZV5, B7NZM0
GO:0006656	BP	phosphatidylcholine biosynthetic process	7	2	0.04	0.00063	G1SZV5, G1SZ91
GO:0007601	BP	visual perception	75	4	0.42	0.00080	G1SKP6, G1SP97, G1T6M1, G1SXP0
GO:0019430	BP	removal of superoxide radicals	8	2	0.04	0.00084	B7NZM0, G1TKH3
GO:0017001	BP	antibiotic catabolic process	8	2	0.04	0.00084	G1TV43, B7NZM0
GO:2000257	BP	regulation of protein activation cascade	8	2	0.04	0.00084	G1SCJ8, G1SIK0
GO:0005615	CC	extracellular space	409	21	2.22	4.0e-14	G1SIK0, G1T4H3, G1SQG6, G1TFV7, G1TFX2, G1STJ4, G1TME7, G1SZV5, B7NZF1, B7NZM0, G1STT3, G1TM88, U3KMR2, G1SDD6, G1SZ91, G1SCJ8, G1TKH3, G1U9S2, G1T7R1, G1TKE4, ...
GO:0019773	CC	proteasome core complex, alpha-subunit c...	7	3	0.04	5.2e-06	G1SWI7, G1T670, G1SZ14
GO:0034361	CC	very-low-density lipoprotein particle	6	2	0.03	0.00043	G1SZV5, B7NZM0
GO:0005839	CC	proteasome core complex	18	5	0.10	0.00138	G1SWI7, G1T670, G1T4X8, G1SZ14, G1T918
GO:0034364	CC	high-density lipoprotein particle	12	2	0.07	0.00185	G1SZV5, B7NZM0
GO:0031252	CC	cell leading edge	111	4	0.60	0.00304	G1TYT3, G1TLY8, G1SWS9, G1U7U3
GO:0120038	CC	plasma membrane bounded cell projection ...	377	7	2.05	0.00408	G1U974, G1TI72, G1U7U3, G1TKH3, G1SSU6, G1SXQ0, G1T918
GO:0030863	CC	cortical cytoskeleton	39	2	0.21	0.01888	B7NZR4, G1TYT3
GO:0005737	CC	cytoplasm	4590	33	24.93	0.02011	G1SLB2, G1SV13, G1SER8, G1TXC0, G1TME7, G1SZD6, G1SZ91, G1SN21, G1T093, G1SKS9, G1T1V5, G1TKH3, G1U9S2, G1SWS9, G1U974, G1T466, G1T671, G1SXQ0, G1T6M1, G1T918, ...
GO:0042824	CC	MHC class I peptide loading complex	5	1	0.03	0.02687	B7NZF1
GO:0005504	MF	fatty acid binding	14	4	0.08	8.9e-07	G1SZ91, G1ST29, G1U9S2, G1T7R1
GO:0042802	MF	identical protein binding	821	17	4.63	2.2e-06	G1SIK0, G1SZV5, B7NZF1, B7NZM0, G1TZP0, G1TEV3,

							G1U7U3, G1SZD6, G1SZ91, G1SN21, G1TKH3, G1TYT3, G1U7S4, G1U9S2, G1SWS9, G1SXQ0, G1TLY8
GO:0003756	MF	protein disulfide isomerase activity	9	3	0.05	1.4e-05	G1T4H3, B7NZF1, G1SFV1
GO:0016868	MF	intramolecular transferase activity, pho...	9	3	0.05	1.4e-05	G1T004, G1SPF5, G1U7S4
GO:0016209	MF	antioxidant activity	38	4	0.21	5.9e-05	G1TKH3, G1SKS9, B7NZM0, G1SQ02
GO:0015037	MF	peptide disulfide oxidoreductase activit...	7	2	0.04	0.00065	G1T4H3, B7NZF1
GO:0008641	MF	ubiquitin-like modifier activating enzym...	10	2	0.06	0.00137	G1SV13, G1T466
GO:0004298	MF	threonine-type endopeptidase activity	13	2	0.07	0.00235	G1T4X8, G1T918
GO:0120020	MF	intermembrane cholesterol transfer activ...	14	2	0.08	0.00273	G1SZV5, B7NZM0
GO:0051087	MF	chaperone binding	50	3	0.28	0.00278	G1T093, G1TKH3, G1U9S2

Table S4. Table with top 10 GO terms for all three GO trees: biological product

(BP), cellular component (CC), and molecular function (MF) for ULu.

GO Tree		Term	Annotated	Significant	Expected	p-value	proteins
GO:0006749	BP	glutathione metabolic process	22	4	0.15	1.5e-05	G1TKH3, G1TD98, G1SXQ0, G1SH63
GO:0006879	BP	cellular iron ion homeostasis	39	4	0.27	0.00015	G1THL2, G1TVS4, G1TKH3, G1STF7
GO:1990748	BP	cellular detoxification	17	3	0.12	0.00021	G1TKH3, G1SXQ0, G1TV43
GO:0031348	BP	negative regulation of defense response	95	5	0.67	0.00053	G1T918, G1SN70, G1SZV5, G1TKH3, G1T2D1
GO:0099563	BP	modification of synaptic structure	7	2	0.05	0.00100	G1SER8, G1T2W2
GO:0052548	BP	regulation of endopeptidase activity	112	5	0.79	0.00113	G1SR03, G1SN70, G1SEK8, G1SEK2, G1SZ14
GO:0022408	BP	negative regulation of cell-cell adhesio...	68	4	0.48	0.00131	G1TYT3, G1SZV5, G1SJI2, G1SEK2
GO:0006098	BP	pentose-phosphate shunt	8	2	0.06	0.00133	G1TAH7, G1T7Z0
GO:0042537	BP	benzene-containing compound metabolic pr...	9	2	0.06	0.00170	G1SXQ0, G1SJI2
GO:0032102	BP	negative regulation of response to exter...	135	5	095	0.00257	G1T918, G1U1Q8, G1SZV5, G1TKH3, G1T2D1
GO:0005615	CC	extracellular space	409	20	2.95		G1SIK0, G1SQG6, G1SNQ8, G1TFX2, G1STJ4, G1TME7, G1SZV5, G1STT3, G1TM88, G1SN70, G1SN67, G1SEK8, U3KMR2, G1SXW8, G1TKH3, G1U9S2, G1U1Q8, G1SI83, G1T7R1, G1STF7, ...
GO:0019773	CC	proteasome core complex, alpha-subunit c...	7	5	0.05	3.5e-10	G1SWI7, G1T519, G1T2L1, G1T670, G1SZ14
GO:0005839	CC	proteasome core complex	18	9	0.13	5.9e-07	G1SWI7, G1SQU1, G1T519, G1T2L1, G1T670, G1T4X8, G1SZ14, G1SHV9, G1T918
GO:0005829	CC	cytosol	1358	24	9.79	1.5e-05	G1T0Z8, G1TET2, G1SJ23, G1SEK2, G1TME7, G1STT3, G1SN70, G1T519, G1T670, G1U7U3, G1T090, G1SN21, G1TRY5, G1SVF3, G1SM24, G1TKH3, G1SWS9, G1U1Q8, G1U974, G1SQ46, ...
GO:0045095	CC	keratin filament	31	4	0.22	6.8e-05	G1SHZ4, G1SHW8, G1U9G5, G1SS18
GO:0016327	CC	apicolateral plasma membrane	8	2	0.06	0.0014	G1SMW6, G1SS18
GO:0005764	CC	lysosome	146	5	1.05	0.0040	G1SJ23, G1SNQ8, G1TAF5, G1SV13, G1SMW6
GO:0045111	CC	intermediate filament cytoskeleton	82	7	0.59	0.0050	G1SS18, G1SWS9, G1SHZ4, G1SHW8, G1U9G5, G1T0Z8, G1SM24
GO:0030139	CC	endocytic vesicle	57	3	0.41	0.0080	G1SZV5, G1SMW6, G1SWS9

GO:0031252	CC	cell leading edge	111	4	0.80	0.0083	G1TET2, G1TYT3, G1SWS9, G1U7U3
GO:0004298	MF	threonine-type endopeptidase activity	13	5	0.09	1.7e-08	G1SQU1, G1T519, G1T4X8, G1SHV9, G1T918
GO:0042802	MF	identical protein binding	821	24	5.65	5.3e-08	G1SIK0, G1TET2, G1SNQ8, G1U8T9, G1SZV5, G1SR28, G1TZP0, G1U7U3, G1SZD6, G1SN21, G1SH63, G1TKH3, G1TYT3, G1TAF5, G1U7S4, G1U9S2, G1SWS9, G1U1Q8, G1SR03, G1SXQ0, ...
GO:0004869	MF	cysteine-type endopeptidase inhibitor ac...	23	4	0.16	1.7e-05	G1TME7, G1SEK8, U3KMR2, G1SN70
GO:0016868	MF	intramolecular transferase activity, pho...	9	3	0.06	2.6e-05	G1T616, G1SPF5, G1U7S4
GO:0002020	MF	protease binding	50	4	0.34	0.00038	G1SIK0, G1TME7, G1SN70, G1SR03
GO:0004364	MF	glutathione transferase activity	21	3	0.14	0.00038	G1SXQ0, G1T0R9, G1TY06
GO:1900750	MF	oligopeptide binding	5	2	0.03	0.00046	G1SH63, G1SXQ0
GO:0003779	MF	actin binding	214	7	1.47	0.00067	G1SER8, G1T823, G1T432, G1SE95, G1TET2, G1TRY5, G1T2C4
GO:0008191	MF	metalloendopeptidase inhibitor activity	8	2	0.06	0.00128	G1SEK8, G1TYE2
GO:0005092	MF	GDP-dissociation inhibitor activity	10	2	0.07	0.00204	G1SNS5, G1SQ46
